# Assessing the impact of gout on cancer risk and the role of healthy lifestyles

**DOI:** 10.3389/fonc.2025.1557175

**Published:** 2025-04-28

**Authors:** Wenru Shi, Jie Zhang, Sitong Wei, Xiang Wang, Hongfei Cao, Dongqing Ye, Xinyu Fang

**Affiliations:** ^1^ Department of Epidemiology and Biostatistics, School of Public Health, Anhui Medical University, Hefei, Anhui, China; ^2^ Inflammation and Immune Mediated Diseases Laboratory of Anhui Province, Hefei, Anhui, China; ^3^ School of Public Health, Anhui University of Science and Technology, Hefei, Anhui, China

**Keywords:** gout, cancer, UK Biobank, health lifestyle, epidemiology

## Abstract

**Background:**

Conflicting evidence exists on the link between gout and cancer risk, with limited clarity on the impact of healthy lifestyle factors.

**Methods:**

In the UK Biobank, 7,169 gout patients were matched with 21,507 non-gout controls (1:3 ratio) using propensity scores. Cox regression models assessed cancer risk associated with gout. Among 6,105 gout patients, cancer risk was further evaluated using an eight-factor Healthy Lifestyle Score (HLS) and a weighted HLS.

**Results:**

Gout was linked to a higher cancer incidence [HR (95% CI) = 1.075 (1.013-1.140)]. High HLS in gout patients correlated with a lower cancer risk [HR (95% CI) = 0.825 (0.717-0.948)], with the strongest protective effect observed in those aged ≥60. Sensitivity analyses confirmed these findings.

**Conclusion:**

Gout patients have a higher risk of developing cancer, but a healthy lifestyle, particularly in those aged 60 and older, significantly reduces this risk. These findings highlight the importance of lifestyle interventions for cancer prevention in patients with gout.

## 1 Introduction

Around 2.5% of the adult population in the UK suffers from gout, the most prevalent inflammatory arthritis in the world (1). It is characterized by an elevation in the concentration of serum uric acid (SUA) in the blood, leading to the deposition of uric acid crystals in tissues such as joints and tendons (2). Hospital admissions for gout have increased by 50-100% in the UK over the past 15–20 years. Despite the alarming increase in hospitalization rates, gout remains frequently underdiagnosed and under-treated, leading to significant losses in work productivity and disability, which in turn considerably escalate healthcare costs and disease burden (2).In recent years, the association between cancer and gout, as well as hyperuricemia, has drawn widespread attention due to their common factor of high cellular turnover, which leads to elevated serum uric acid (SUA) levels. This elevation is seen in conditions like hemolysis, tumor lysis syndrome, and cancer, underscoring these conditions’ interconnected nature (3, 4). Previous studies have suggested that SUA functions as an inhibitor of reactive oxygen species formation and therefore has some anticancer protective effects (5), but some studies have found SUA to be associated with pro-inflammatory mediators that may promote cancer development (6). Moreover, the results of epidemiological studies remain controversial. Elevated SUA levels have been independently linked to higher cancer risk in some studies (7, 8). However, other studies have indicated that elevated SUA is associated with reduced cancer mortality (9, 10). Furthermore, although emerging studies have explored the relationship between gout and cancer, the connection between these two conditions has not been extensively studied or deeply understood. Studies, such as those conducted in Taiwan by Kuo et al. (11) and in Korea by Oh et al. (12), have suggested an increased occurrence of cancer in gout patients compared to control groups. However, these studies frequently neglect the complex influence of comorbidities prevalent among gout patients, such as hypertension, chronic kidney disease (CKD), obesity, and cardiovascular diseases (CVD), each of which independently increases the risk of cancer (13). The failure to adequately account for these comorbidities and to closely examine SUA levels means that the understanding of the gout-cancer relationship is still insufficient.

In addition, lifestyle is a modifiable factor that can reduce cancer risk. More than 40% of all cancer cases and deaths are attributed to potentially modifiable risk factors, mainly stemming from unhealthy lifestyles (14). Several lifestyle factors that can be modified have been identified as cancer risk factors in healthy populations. These risk factors include unhealthy weight (15), cigarette smoking (16), heavy alcohol consumption (17), low physical activity (18), low vegetable and fruit intake (19), high red meat intake (20), Unreasonable sleep schedule (21). Given the common coexistence of lifestyle factors, researchers have found associations between various combinations of these factors and cancer risk in numerous recent studies, providing partial evidence that an overall healthy lifestyle is linked to reduced cancer risk (22, 23). Sex differences in lifestyle behaviors, such as diet and activity, also influence cancer susceptibility, affecting men and women differently (24, 25). However, the combined impact of these factors on cancer risk in gout patients has not been systematically studied.

Therefore, this prospective, large-scale, population-based study using UKB data aims to assess whether gout increases cancer risk by considering the role of uric acid levels and accounting for the effects of concomitant diseases, in addition to evaluating if a healthy lifestyle can mitigate this risk among gout patients. This will help uncover the true cancer risk for gout patients, thereby providing a foundation for clinical management and prevention strategies.

## 2 Methods

### 2.1 Study design and participants

This study was based on the UK Biobank, with data collection and follow-up spanning from 2006 to 2022. At baseline (2006-2010), participants were recruited, and comprehensive data on demographics, lifestyle behaviors, medical history, and blood biomarkers were collected. Gout cases were identified from baseline through 2022 using hospital inpatient records (Hospital Episode Statistics, HES), general practitioner (GP) records, and self-reported diagnoses. Cancer outcomes were tracked until December 31, 2022, through the National Cancer Registration and Analysis Service (NCRAS), which provided information on cancer diagnosis dates, types, and ICD-10 codes. Mortality data were obtained from the Office for National Statistics (ONS), ensuring complete follow-up on deaths occurring up to December 31, 2022. Comprehensive descriptions of the study design and protocol are available in other publications (26). The UK Biobank study received approval from the North West Multicenter Research Ethical Committee (11/NW/0382), and all participants provided written informed consent.

Participants with confirmed or self-reported gout, as indicated by International Classification of Diseases, 10th Revision (ICD-10) codes M10.0–M10.9, recorded by hospitalization records or self-reported use of allopurinol and sulfinpyrazone medications, were specifically recruited for this study (**Supplementary Table S1**). Subsequently, we excluded 46,128 participants who had been diagnosed with cancer before or on the day of enrollment. In addition, participants who were lost to follow-up were excluded (N = 1297). After 1:3 propensity score matching based on age and sex, a total of 28,676 participants (7,169 with gout and 21,507 without gout) were included to analyze the association between gout and cancer risk. On this basis, we further excluded 1,047 participants with missing lifestyle data and 17 individuals who were excluded due to time logic errors, and finally included 6,105 gout patients to evaluate the impact of lifestyle scores on cancer risk in the gout population (**Supplementary Figure S1**).

### 2.2 Definition of health lifestyle score

In this study, the selection of healthy lifestyle score (HLS) components was based on the World Cancer Research Fund/American Institute for Cancer Research (WCRF/AICR) recommendations and previous epidemiological evidence linking lifestyle factors to cancer risk. The WCRF/AICR guidelines emphasize the importance of maintaining a healthy weight, engaging in regular physical activity, limiting alcohol consumption, avoiding smoking, and adhering to a balanced diet rich in plant-based foods to reduce cancer risk (27). Therefore, we constructed the HLS using key modifiable lifestyle factors, including the following: waist circumference (WC) and body mass index (BMI), sedentary time (time spent on activities such as computer use, watching television, and driving) (28), physical activity level, fruit and vegetable intake, grain intake, red meat intake, alcohol frequency, smoking status, and sleep duration (29). These factors have been associated with both gout and cancer risk (30, 31). Each component was assigned a score based on adherence to established public health guidelines, with higher scores indicating healthier behaviors. In addition, gender differences in lifestyle behaviors may influence the associations between various lifestyle behaviors and cancer risk. Thus, two HLSs were developed: an unweighted HLS and a gender-specific weighted HLS. Each factor was scored on a scale from 0 to 1, with 1 indicating the healthiest behavioral category (**Supplementary Table S2**). The scores of the eight lifestyle factors were summed to generate the unweighted HLS, ranging from 0 to 8. This HLS was analyzed across three categories: low (< 2.75), medium [2.75, 3.75), and high (≥ 3.75), based on the tertile distribution among all participants (32). To provide a more detailed reflection of each lifestyle behavior, we developed gender-specific weighted HLSs. These were derived from the β coefficients for each lifestyle factor in the Cox proportional risk regression model, stratified by gender. In this study, men were classified as low (≥ -0.374), medium [-0.552, -0.374), and high (< -0.552) based on the three-digit distribution of weighted HLS, while women were categorized as low (≥ -0.219), medium [-0.469, -0.219), and high (< -0.469) (33).

### 2.3 Outcomes ascertainment

The study focused on incident cancer events as the primary outcomes of interest. These events were identified through self-reported information and linked to various health-related records, including primary care data, hospital admissions, cancer registries, and death registration system, as provided by the UK Biobank. Participants were followed from enrollment until the earliest event of interest, which could include the outcomes, death, loss to follow-up, or the end of the follow-up period. Admission data were available until October 31, 2022, and mortality data until December 31, 2022.

### 2.4 Covariates

The selection of covariates was based on previous analyses of the literature to account for potential confounders of the association between gout and cancer risk. The main covariates included age (in years), sex, ethnicity (classified as white or non-white), education level (college, high school, middle school, or vocational/other), and socioeconomic status, assessed using the Townsend Deprivation Index, a validated measure of socioeconomic deprivation in the United Kingdom (34). Comorbidities were also considered as major confounders. Physician-diagnosed vascular and cardiac diseases (including hypertension, angina, stroke, and heart attack) were also included, as these diseases have been associated with gout and cancer risk through shared inflammatory and metabolic pathways (35, 36). Diabetes, another major metabolic disorder, was also adjusted for due to its established association with gout and cancer risk (2). In addition, uric acid levels collected from blood samples at study recruitment were included as potential biological factors influencing the relationship between gout and cancer (**Supplementary Table S3**).

### 2.5 Statistical analysis

Statistical analyses were conducted using R software version 4.3.3, with a significance level of 0.05 (two-sided). Kaplan-Meier methods generated cumulative cancer incidence curves, and Cox proportional hazards models estimated hazard ratios (HR) and 95% confidence intervals (CI) for the association between gout and cancer, adjusting for race, socioeconomic status, education, smoking, alcohol consumption, urate levels, and BMI. Missing covariate data were handled through multiple imputations with five replications using a chained-equation approach (37) (**Supplementary Table S15**). The proportional hazards assumption of the Cox model was tested using the Schoenfeld residual method, and no violation of this assumption was observed. We further employed Cox models to assess the impact of lifestyle factors on cancer incidence in gout patients, adjusting for age, sex, ethnicity, education, socioeconomic status, urate levels, and comorbidities (e.g., high blood pressure, angina, stroke, heart attack, diabetes). Participants were grouped by age (<60 and ≥60), sex, and urate levels to explore these factors’ influence on the lifestyle-cancer link. Sensitivity analyses were performed, excluding cancer cases within two years to avoid reverse causality. We also applied Fine and Gray’s sub-distribution method to account for competing risks, including death, and examined the effects of HLS and weighted HLS on cancer incidence over short (≤5 years), intermediate (≤10 years), and long-term (≤15 years) follow-up periods (38).

## 3 Results

### 3.1 Baseline population characteristics

The initial segment of this study encompassed 7169 UK Biobank participants diagnosed with gout and devoid of cancer, alongside 21,507 UK Biobank participants without gout and cancer. The average age of participants was 59.6 (SD = 7.0) years, with a majority being men (93.0%). During the follow-up (12.08 ± 3.37 years), 6157 incident cancer cases were identified. Compared to those without gout, individuals with gout had lower education levels, a higher prevalence of low socio-economic status, increased smoking and alcohol consumption rates, and higher BMI and urate levels (**Table 1**). Among those with gout, variations in lifestyle factors are observed across genders, while additional baseline characteristics are detailed in the Appendix for further context (**Supplementary Tables S5-S7**).

**Table 1:** Baseline characteristics of patients with and without gout.

Characteristics	Part A^a^			Part B^b^
	Gout group (n=7169)	Non-gout group (n=21507)	*P* value	Gout group (n=6105)
Cancer			0.132	
No	4392	17115		4752 (0.78)
Yes	1585	5584		1353 (0.22)
Age, years, mean (SD)	59.6 (7.00)	59.6 (7.00)	0.988	59.4 (7.00)
Sex			0.968	
Female	506	1518		377 (0.06)
Male	6663	19989		5728 (0.94)
Race			0.121	
White	6790	20449		5862 (0.96)
Non-white	379	1058		243 (0.04)
Educational level			<0.001	
College or University	1761	6891		1652 (0.27)
Upper secondary	715	2056		662 (0.11)
Lower secondary	1779	4833		1584 (0.26)
Vocational or other	2873	7554		2207 (0.36)
Socio-economic status			<0.001	
Low	2555	6980		2030 (0.33)
Middle	2376	716		2036 (0.33)
High	2238	7365		2039 (0.34)
Smoking status			<0.001	
Never	3054	10336		2607 (0.43)
Previous	3449	8755		2950 (0.48)
Current	660	2381		548 (0.09)
Alcohol intake frequency			<0.001	
Daily or almost daily	2350	5641		2069 (0.34)
Three or four times a week	1946	5534		1698 (0.28)
Once or twice a week	1632	5298		1369 (0.23)
One to three times a month	435	1862		362 (0.06)
Special occasions only	416	1695		311 (0.05)
Never	394	1442		296 (0.05)
BMI, kg/m^2^, mean (SD)	30.80 (4.99)	28.50 (4.53)	<0.001	30.66 (4.90)
Vascular/heart problems			0.874	
Heart attack	542	903		433 (0.07)
Angina	391	769		320 (0.05)
Stroke	205	368		168 (0.03)
High blood pressure	3322	5917		2844 (0.47)
Diabetes	1036	1546	0.227	843 (0.14)
Urate, umol/L, mean (SD)	379.0 (102.0)	355.0 (76.0)	<0.001	379.96 (102.47)

a is exploring whether gout can increase cancer risk;

b is exploring whether a healthy lifestyle can reduce the increased cancer risk of gout.

### 3.2 The cumulative incidence of overall cancer in patients with and without gout

**Figure 1:**
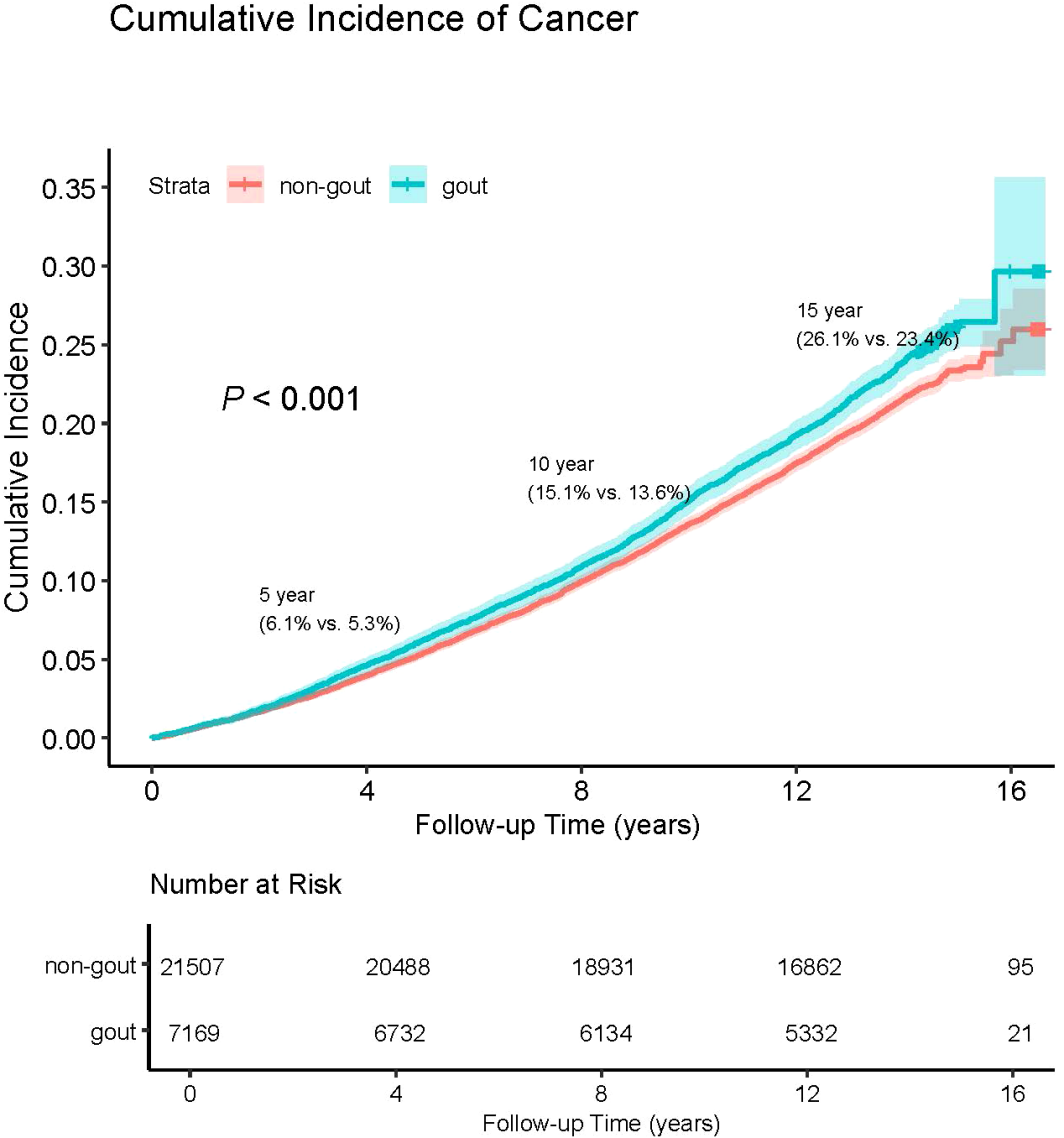
Cumulative incidence of overall cancer in patients with and without gout.

The crude cancer incidence rate ratio between gout patients and non-gout patients in this study was 1.114. This highlights the cumulative hazard risks of overall cancer in both gout and non-gout patients (**Figure 1**). The Cox proportional hazards regression analysis revealed that gout patients had a higher risk of developing cancer compared to non-gout patients [HR (95% CI) = 1.122 (1.060-1.189)]. This association remained significant even after adjusting for basic demographic characteristics and other covariates [HR (95% CI) = 1.087(1.020-1.158)] (**Supplementary Table S4**).

### 3.3 Influence of healthy lifestyle on cancer incidence in individuals with gout

**Figure 2:**
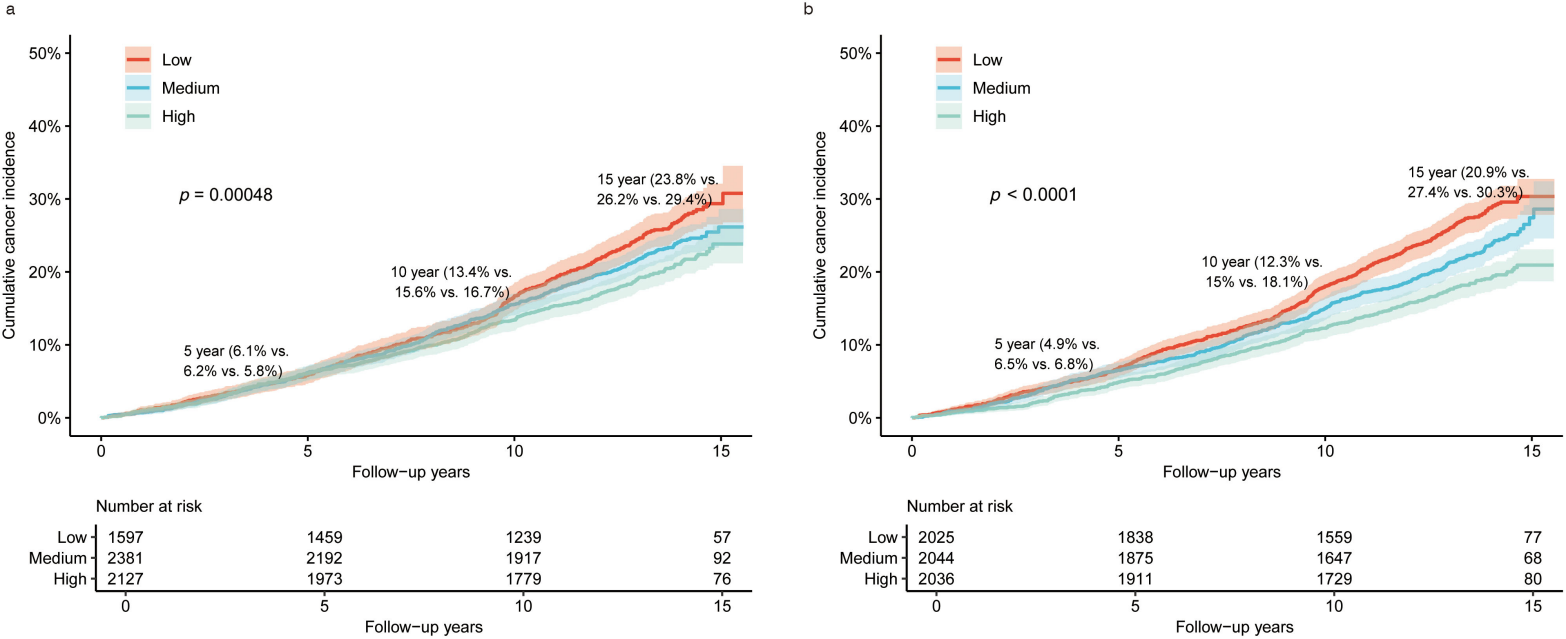
Overall cumulative cancer incidence in the gout population under different categories of HLS and weighted HLS. **(a)** is the overall cancer incidence in the gout population under different categories of HLS; **(b)** is the overall cumulative cancer incidence rate in the gout population under different categories of weighted HLS.

After adjusting for covariates, individuals who adhered to a high HLS had a decreased risk of cancer compared with those with a low HLS [HR (95% CI) = 0.825(0.717-0.948)]. Similarly, both medium-weighted HLS [HR (95% CI) = 0.853(0.751-0.967)] and high-weighted HLS [HR (95% CI) = 0.790(0.690-0.905)] were significantly associated with reduced cancer risk relative to low-weighted HLS (**Table 2**). **Figure 2** illustrates the cumulative risk of cancer development over the follow-up period for both the unweighted and weighted HLS groups. It’s evident that the risk of cancer is higher in the low HLS group and lowest in the high HLS group, with a more pronounced trend observed in the weighted HLS. In the Cox regression model, four out of the eight examined lifestyle factors were associated with a decreased risk of cancer: lower red meat intake [HR (95% CI) = 0.742(0.574-0.959)], adequate sleep duration [HR (95% CI) = 0.814(0.695-0.953)], moderate alcohol consumption [HR (95% CI) = 0.833(0.705-0.985)] and non-smoking [HR (95% CI) = 0.716(0.590-0.868)] (**Table 2**).

**Table 2:** Basic and multiple adjusted hazard ratios (HR) and 95% confidence intervals (CI) for cancer by lifestyle factors in a gouty population: results from one-way Cox regression modeling.

	Cancer/noncancer	Model 1^a^		*P* trend	Model 2^b^	
		HR (95% CI)	*P-*value		HR (95% CI)	*P-*value
BMI (kg/m^2^)				0.112		
Unfavorable	621/2327	1.000 (ref)			1.000 (ref)	
Intermediate	617/2027	1.105 (0.9881.236)	0.078		1.072 (0.956,1.202)	0.232
Favorable	115/398	1.091 (0.894,1.332)	0.389		1.085 (0.885,1.329)	0.430
WC (cm)				0.762		
Unfavorable	742/2649	1.000 (ref)			1.000 (ref)	
Intermediate	365/1257	1.024 (0.903,1.161)	0.712		1.034 (0.910,1.175)	0.605
Favorable	246/846	1.016 (0.879,1.174)	0.827		1.038 (0.896,1.204)	0.613
Physical activity 10+ min (days/week)				0.885		
Unfavorable	220/750	1.000 (ref)			1.000 (ref)	
Intermediate	587/2143	0.927 (0.794,1.083)	0.342		0.905 (0.774,1.058)	0.212
Favorable	546/1859	0.983 (0.840,1.150)	0.831		0.946 (0.807,1.107)	0.491
Sedentary time (hours/day)				0.192		
Unfavorable	851/2913	1.000 (ref)			1.000 (ref)	
Intermediate	399/1446	0.943 (0.837,1.063)	0.339		0.965 (0.856,1.088)	0.561
Favorable	103/393	0.896 (0.730,1.099)	0.293		0.926 (0.753,1.139)	0.471
Fruit and vegetable intake (servings/day)				0.596		
Unfavorable	500/1782	1.000 (ref)			1.000 (ref)	
Intermediate	500/1749	1.014 (0.895,1.138)	0.828		0.916 (0.809,1.038)	0.173
Favorable	353/1221	1.038 (0.905,1.189)	0.592		0.925 (0.806,1.061)	0.267
Whole grains intake (servings/day)				0.409		
Unfavorable	428/1581	1.000 (ref)			1.000 (ref)	
Intermediate	885/3018	1.077 (0.960,1.209)	0.205		1.005 (0.894,1.129)	0.928
Favorable	40/153	0.943 (0.682,1.305)	0.727		0.951 (0.686,1.319)	0.766
Meat intake (times/week)				0.021		
Unfavorable	119/380	1.000 (ref)			1.000 (ref)	
Intermediate	669/2398	0.900 (0.745,1.087)	0.277		0.954 (0.789,1.153)	0.629
Favorable	189/780	0.742 (0.574,0.959)	0.023		0.849 (0.655,1.099)	0.214
Alcohol intake frequency				0.015		
Unfavorable	132/416	1.000 (ref)			1.000 (ref)	
Intermediate	742/2208	0.885 (0.788,0.994)	0.039		0.951 (0.845,1.069)	0.403
Favorable	479/2128	0.833 (0.705,0.985)	0.033		0.920 (0.771,1.096)	0.351
Smoking				<0.001		
Unfavorable	230/720	1.000 (ref)			1.000 (ref)	
Intermediate	650/2255	1.042 (0.866,1.254)	0.657		0.804 (0.666,0.970)	0.023
Favorable	473/1777	0.716 (0.590,0.868)	<0.001		0.676 (0.556,0.821)	<0.001
Sleep time (hours/day)				0.012		
Unfavorable	397/1200	1.000 (ref)			1.000 (ref)	
Intermediate	532/1849	0.877 (0.754,1.020)	0.088		0.957 (0.822,1.113)	0.570
Favorable	424/1703	0.814 (0.695,0.953)	0.010		0.956 (0.814,1.122)	0.585
HLS				<0.001		
Low	397/1200	1.000 (ref)			1.000 (ref)	
Medium	532/1849	0.874 (0.767,0.995)	0.043		0.890 (0.781,1.015)	0.082
High	424/1703	0.761 (0.664,0.873)	<0.001		0.825 (0.717,0.948)	0.006
Weighted HLS				<0.001		
Low	532/1493	1.000 (ref)			1.000 (ref)	
Medium	450/1594	0.812 (0.716,0.920)	<0.001		0.853 (0.751,0.967)	0.013
High	371/1665	0.643 (0.563,0.734)	<0.001		0.790 (0.690,0.905)	<0.001

a Model 1 was not adjusted;

b Model 2 was adjusted for age, sex, race, education level, Thomson index, heart/cardiovascular disease, diabetes mellitus, and urate.

### 3.4 Uric acid-linked lifestyle factors and cancer risk in gout patients

To assess if urate levels influenced the association between lifestyle factors and cancer risk, we conducted further analyses by stratifying based on urate levels (**Supplementary Table S8**). In the stratified analysis, unweighted HLS showed differential associations with cancer risk across different urate-level groups. Specifically, high HLS [HR (95% CI) = 0.643(0.497-0.832)] exhibited a trend towards significantly lower cancer risk in the high urate level groups. Our study revealed that individuals with higher urate levels tended to adhere more closely to lifestyle recommendations, and inversely, exhibited a lower cancer risk. In both the low and medium urate level groups, both moderate and high-weighted HLS were significantly linked to reduced cancer risk. However, after adjusting for covariates, only high-weighted HLS were significantly associated with lower cancer risk. However, in the high uric acid level group, only the highly weighted HLS showed a significant association with reduced cancer risk. Nevertheless, this association disappeared after adjusting for covariates.

### 3.5 Subgroup analysis

In a gender-stratified analysis of gout patients, adopting a higher level of healthy living was linked to reduced cancer risk in men [HR (95% CI) = 0.752(0.653-0.867)]. In both men [HR (95% CI) = 0.816(0.710-0.938)] and women [HR (95% CI) = 0.482(0.262-0.887)], a high weighted HLS was associated with a reduced risk of cancer development (**Supplementary Tables S9**, **S10**). We additionally examined the influence of age on the relationship between HLS and cancer risk (**Supplementary Table S11**). Among individuals aged 60 years and older, the findings demonstrated that the high lifestyle group had a lower cancer risk compared to the low lifestyle group [HR (95% CI) = 0.786(0.670-0.922)]. Moreover, both medium [HR (95% CI) = 0.834(0.723-0.960)] and high weighted HLS [HR (95% CI) = 0.734(0.625-0.860)] were associated with reduced cancer risks compared to the low weighted HLS group.

### 3.6 Sensitivity analysis

After excluding participants who died or developed cancer within the initial two years of the study (5967 participants remaining), there was a more pronounced trend toward lower cancer risk among those with higher healthy lifestyle adherence than among those with lower adherence in both unweighted and weighted HLS. These results remain consistent even after controlling for potential confounders (**Supplementary Table S12**). The competing risks regression analysis model indicated a more pronounced trend of cancer risk reduction in high-weighted HLS compared to low-weighted HLS after adjusting for the competing relationship between cancer events and mortality [HR (95% CI) = 0.866(0.766-0.978)]. This suggests that lifestyle factors may play a crucial role in reducing cancer risk in gout populations (**Supplementary Table S13**). In our study, we focused on analyzing the effect of HLS/weighted HLS on different survival time stages (≤5 years, ≤10 years, ≤15 years). The findings suggest that the adoption of a high level of weighted healthy lifestyle may have a significant benefit in prolonging survival time during the long-term survival time phase, especially at ≤15 years (**Supplementary Table S14**).

## 4 Discussion

In this extensive prospective study, we observed that the cancer incidence rate in the gout group was 1.059 times higher than in the non-gout group over a follow-up period of 12.08 years. Our results showed that gout patients had a 7.5% higher risk of cancer after adjusting for covariates. Furthermore, adopting a healthier lifestyle was linked to a decreased cancer risk among gout patients. Specifically, following a high-quality healthy lifestyle was linked with a reduction in cancer risk ranging from approximately 17.5% to 23.9%. Additionally, maintaining a healthy lifestyle was associated with a decreased risk of cancer, irrespective of the participant’s age, gender, or urate levels. These findings remained consistent even after excluding data with less than two years of follow-up and applying competing risk regression models.

Previous studies have suggested a positive correlation between gout and cancer. For example, In a Swedish study (39), it was reported that the incidence of cancer among individuals with gout was 1.25 times higher than that of the general population. The result of our study was 1.11. Although this risk ratio was low, it may be related to factors such as different gout patient characteristics, sample size, and follow-up time in the study. Additionally, a Korean study of a middle-aged cohort by Lee et al. (40) observed that compared to the general population, middle-aged patients with gout had significantly higher risks of cancer, all-cause mortality, and cancer-specific mortality. These findings are consistent with our study, which found that even after adjusting for comorbidities like diabetes, hypertension, angina, stroke, heart attack, and uric acid levels, individuals with gout still had a significantly higher risk of cancer, suggesting gout may be a potential cancer risk factor.

While the exact mechanisms linking gout to an increased cancer risk remain unclear, several lines of evidence suggest possible pathways. First, IL-1β not only triggers acute inflammatory responses but also promotes chronic inflammation, a well-known cancer risk factor (41). Chronic inflammation can alter the local tissue microenvironment, leading to fibrosis and tissue remodeling which might create a pro-tumorigenic niche facilitating the initiation and progression of malignant cells (42). Furthermore, chronic inflammation is linked to heightened production of reactive oxygen species (ROS), which induces oxidative stress, potentially resulting in DNA damage, genomic instability, and mutations—key processes in carcinogenesis (43). Furthermore, hyperuricemia, a hallmark of gout, contributes to metabolic dysregulation, impacting insulin resistance, obesity, and dyslipidemia, which are recognized as cancer risk factors, particularly affecting the liver, pancreas, and colorectal regions (44). Elevated uric acid levels themselves may also contribute directly to oxidative stress and inflammation, exacerbating cancer risks (45).

The interaction between gout and cancer is further complicated by lifestyle factors common to both diseases. Previous studies indicate that approximately 37.7% of cancers in the UK could be prevented annually through lifestyle modifications (46). Following a healthy lifestyle is known to lower overall cancer risk (47), though studies have largely focused on healthy populations, with few examining gout patients specifically. Our study found an inverse relationship between high HLS and cancer incidence in gout patients, which remained consistent after adjusting for covariates and across sensitivity analyses, including exclusions for early cancer cases and competing mortality risks. This result may be affected by individual metabolic differences, and the reduced uric acid levels in some individuals with low uric acid levels may be due to chronic diseases or malnutrition, rather than the protective or promoting effect of uric acid itself on cancer risk. In addition, the inherent limitations of observational studies, especially the limitations of causal inference, may affect our interpretation of this association. Although the contribution of different factors to cancer may vary, specific lifestyle factors - sleep duration, red meat intake, alcohol consumption and smoking - are consistent with previous research results (16, 17, 20, 21), which are generally consistent with the World Cancer Research Fund/American Institute for Cancer Research (WCRF/AICR) recommendations. The combined effects of lifestyle factors are more significant. As the literature indicates, the combined effects of multiple health behaviors are often more significant in affecting cancer risk (48). At higher uric acid levels, patients with high HLS showed reduced cancer risk, unlike prior studies that found little association, possibly due to healthy volunteer bias. One important aspect of our study is the predominantly male cohort (93% male participants), reflecting the higher gout prevalence in men. While the HLS-cancer association remained significant in sex-stratified analyses, the effect size appeared weaker in women, possibly due to the smaller sample size and differences in hormonal and metabolic factors that influence cancer susceptibility (35). Larger female cohort studies are needed to confirm these associations and strengthen external validity. Our results suggest that the protective effect of a higher healthy lifestyle score (HLS) on cancer risk is most pronounced in older adults (≥60 years old). This may be attributed to age-related metabolic and physiological changes, in which healthy behaviors help offset oxidative stress, chronic inflammation, and decreased immunity associated with aging (49). In addition, health selection bias may also play a role, as older adults who maintain a healthy lifestyle may represent a subset with better baseline health status.

This study has several strengths, including a large sample size, the prospective design of the UK Biobank, and a thorough evaluation of cancer risk among gout patients. It is the first to focus on how a healthy lifestyle impacts cancer risk in this population. However, several limitations should be acknowledged. Lifestyle factors were self-reported, which may introduce misclassification, and were assessed only at baseline, potentially overlooking subsequent behavioral changes. The limited representation of women may restrict the generalizability of our findings to female populations. Additionally, while we examined overall cancer risk, associations with specific cancer types require further investigation. Although we adjusted for common gout comorbidities such as hypertension, obesity, and cardiovascular disease, we cannot exclude the potential effects of other unmeasured diseases and lifestyle factors.

## 5 Conclusion

In summary, our study reveals that individuals with gout face a higher risk of cancer. However, adopting a healthier lifestyle can significantly mitigate this risk, highlighting the importance of lifestyle modifications in reducing cancer risk among gout patients.

## Data Availability

The datasets presented in this study can be found in online repositories. The names of the repository/repositories and accession number(s) can be found below: UK Biobank data can be requested by researchers for approved projects, including replication, through https://www.ukbiobank.ac.uk/.
