# Supplementary material for: Assessing the impact of gout on cancer risk and the role of healthy lifestyles
Source: Front Oncol. 2025 Apr 28;15:1557175. doi: 10.3389/fonc.2025.1557175 (PMC12066266; doi:10.3389/fonc.2025.1557175)
Supplement: Supplementary file 1 [file DataSheet1.docx]

**Assessing the impact of gout on cancer risk and the role of healthy lifestyles: the UK Biobank population study**

This study strictly adheres to the STROBE Statement—Checklist of items that should be included in reports of cohort studies, ensuring transparency and standardization in study design, data analysis, and result reporting as follows.

|  | Item No | Recommendation | Page No | |
| --- | --- | --- | --- | --- |
| **Title and abstract** | 1 | (*a*) Indicate the study’s design with a commonly used term in the title or the abstract | 1 | |
|  |  | (*b*) Provide in the abstract an informative and balanced summary of what was done and what was found | 2 | |
| Introduction | | |  |  |
| Background/rationale | 2 | Explain the scientific background and rationale for the investigation being reported | 4-5 | |
| Objectives | 3 | State specific objectives, including any prespecified hypotheses | 4-5 | |
| Methods | | |  |  |
| Study design | 4 | Present key elements of study design early in the paper | 5-7 | |
| Setting | 5 | Describe the setting, locations, and relevant dates, including periods of recruitment, exposure, follow-up, and data collection | 5-7 | |
| Participants | 6 | (*a*) Give the eligibility criteria, and the sources and methods of selection of participants. Describe methods of follow-up | 5-7 | |
|  |  | (*b*) For matched studies, give matching criteria and number of exposed and unexposed | 5-7 | |
| Variables | 7 | Clearly define all outcomes, exposures, predictors, potential confounders, and effect modifiers. Give diagnostic criteria, if applicable | 5-7 | |
| Data sources/ measurement | 8* | For each variable of interest, give sources of data and details of methods of assessment (measurement). Describe comparability of assessment methods if there is more than one group | 5-7 | |
| Bias | 9 | Describe any efforts to address potential sources of bias | 5-7 | |
| Study size | 10 | Explain how the study size was arrived at | 5-7 | |
| Quantitative variables | 11 | Explain how quantitative variables were handled in the analyses. If applicable, describe which groupings were chosen and why | 5-7 | |
| Statistical methods | 12 | (*a*) Describe all statistical methods, including those used to control for confounding | 5-7 | |
|  |  | (*b*) Describe any methods used to examine subgroups and interactions | 5-7 | |
|  |  | (*c*) Explain how missing data were addressed | 5-7 | |
|  |  | (*d*) If applicable, explain how loss to follow-up was addressed | 5-7 | |
|  |  | (*e*) Describe any sensitivity analyses | 5-7 | |
| Results | | |  | |
| Participants | 13* | (a) Report numbers of individuals at each stage of study—eg numbers potentially eligible, examined for eligibility, confirmed eligible, included in the study, completing follow-up, and analysed | 7 | |
|  |  | (b) Give reasons for non-participation at each stage | 7 | |
|  |  | (c) Consider use of a flow diagram | 7 | |
| Descriptive data | 14* | (a) Give characteristics of study participants (eg demographic, clinical, social) and information on exposures and potential confounders | 7-10 | |
|  |  | (b) Indicate number of participants with missing data for each variable of interest | 7-10 | |
|  |  | (c) Summarise follow-up time (eg, average and total amount) | 7-10 | |
| Outcome data | 15* | Report numbers of outcome events or summary measures over time | 7-10 | |
| Main results | 16 | (*a*) Give unadjusted estimates and, if applicable, confounder-adjusted estimates and their precision (eg, 95% confidence interval). Make clear which confounders were adjusted for and why they were included | 7-10 |  |
|  |  | (*b*) Report category boundaries when continuous variables were categorized | 7-10 |  |
|  |  | (*c*) If relevant, consider translating estimates of relative risk into absolute risk for a meaningful time period | 7-10 |  |
| Other analyses | 17 | Report other analyses done—eg analyses of subgroups and interactions, and sensitivity analyses | 7-10 |  |
| Discussion | |  |  |  |
| Key results | 18 | Summarise key results with reference to study objectives | 10-12 |  |
| Limitations | 19 | Discuss limitations of the study, taking into account sources of potential bias or imprecision. Discuss both direction and magnitude of any potential bias | 10-12 |  |
| Interpretation | 20 | Give a cautious overall interpretation of results considering objectives, limitations, multiplicity of analyses, results from similar studies, and other relevant evidence | 10-12 |  |
| Generalisability | 21 | Discuss the generalisability (external validity) of the study results | 10-12 |  |
| Other information | |  |  |  |
| Funding | 22 | Give the source of funding and the role of the funders for the present study and, if applicable, for the original study on which the present article is based | 13 |  |

*Give information separately for exposed and unexposed groups.

**Note:** An Explanation and Elaboration article discusses each checklist item and gives methodological background and published examples of transparent reporting. The STROBE checklist is best used in conjunction with this article (freely available on the Web sites of PLoS Medicine at http://www.plosmedicine.org/, Annals of Internal Medicine at http://www.annals.org/, and Epidemiology at http://www.epidem.com/). Information on the STROBE Initiative is available at http://www.strobe-statement.org.

In the propensity score matching (PSM) analysis, the index date was defined as follows. For gout patients (exposed group), the index date was set as the date of first gout diagnosis, identified through hospital inpatient records, general practitioner (GP) records, or self-reported diagnoses. For non-gout controls (matched at a 1:3 ratio), the index date was aligned with the corresponding gout patient's diagnosis date to ensure a consistent observation starting point and minimize temporal bias. To confirm the temporal alignment between the matched groups, we assessed the distribution of index dates in both groups and conducted statistical tests (t-test or chi-square test). The results indicated no significant difference in index dates between the gout and non-gout groups (*P* > 0.05), ensuring comparability in baseline time points.

To ensure the validity of the Cox proportional hazards model, we tested the proportional hazards (pH) assumption using Schoenfeld residuals. All individual covariates had P-values greater than 0.05, indicating no significant violation of the proportional hazards assumption. Additionally, visual inspection of Schoenfeld residual plots showed no clear patterns suggesting time-dependent effects. These results confirm that the Cox model is appropriate for analyzing the association between gout and cancer risk(Figure S2).

Principal Component Analysis (PCA) was applied to determine data-driven weights for each Healthy Lifestyle Score (HLS) component. The first principal component (PC1) was extracted, and absolute loadings were normalized to obtain variable weights. The final weighted HLS was calculated as a linear combination of standardized lifestyle factors. The highest contributing factors were BMI (29.4%) and waist circumference (29.5%), followed by sedentary behavior (12.9%) and physical activity (9.8%). Smoking had the lowest weight (<1%), suggesting a limited role in explaining variance in HLS among our study population. The PCA-derived HLS classification remained robust across different weighting methods (Table S15).

Figure S3 shows the VIF value of each variable. The results show that the VIF values ​​of all variables are low, indicating that the multicollinearity problem between them is not significant. It can be seen that there is no serious collinearity between the variables in the regression model, which further supports the reliability of the research results.

**Supplementary Tables**

Table S1 Codes used in the UK Biobank study to identify gout cases

Table S2 Unweighted healthy lifestyle score components

Table S3 Data fields and information on variables in the UK Biobank cohort involved in this study

Table S4 HR for cancers in patients with and without gout (controls) after adjusting for covariates

Table S5 Baseline characteristics of participants in the UK Biobank classified by the presence or absence of cancer

Table S6 Baseline characteristics of lifestyle factors among gout patients by gender (n=6105)

Table S7 Baseline characteristics of gout patients by unweighted and weighted HLS classification (n=6105)

Table S8 Results of one-way Cox regression analysis of lifestyle factors and cancer incidence in gout patients stratified by urate level

Table S9 Results of one-way Cox regression analysis of lifestyle factors and cancer incidence in male gout patients

Table S10 Results of one-way Cox regression analysis of lifestyle factors and cancer incidence in female gout patients

Table S11 Results of one-way Cox regression analysis of gout patients stratified by age due to lifestyle factors and cancer occurrence

Table S12 Relationship between lifestyle and cancer risk in gout patients after removal of follow-up time of less than 2 years

Table S13 Results of competing risk regression analyses of lifestyle and cancer risk in gout patients

Table S14 Impact of HLS and weighted HLS on survival time at short-, medium- and long-term follow-up times

Table S15 Number and percentage of missing values in interpolated data variables

**Supplementary Figure**

Figure S1 Flowchart of Participant Selection

Figure S2 Schoenfeld residual plot

Figure S3 Scatter Plot of VlF with Variables

**Table S1 Codes used in the UK Biobank study to identify gout cases.**

| **Source** | **UK Biobank data fields** | **Corresponding codes** |
| --- | --- | --- |
| **Hospital inpatient data**  Gout diagnosis of ICD-10 | 41720: Diagnosis - ICD10 | **ICD-10**:M10.0,M10.1,M10.2,M10.3,M10.4,M10.9 |
| **Medical conditions**  Self-report past and current Gout through a verbal interview by a trained nurse. | 20002: Non-cancer illness code, self-reported | 1466 |
| **Prescription medication**  Self-report past and current Gout-relevant medicine taken through a verbal interview by a trained nurse. | 20003: Treatment/medication code | 1140875408, 1140909890 |

**Table S2 Unweighted healthy lifestyle score components**

| Recommendations ^a^ | Detailed information | Categories | Score |
| --- | --- | --- | --- |
| 1. Be a healthy weight | BMI ^b^(kg/m^2^) |  |  |
|  | ≥18.5-<25.0  25.0-29.9  <18.5 or ≥30 | Favorable  Intermediate  Unfavorable | 0.5  0.25  0 |
|  | Waist circumference (cm) |  |  |
|  | Men: <94  Women: <80  Men: 94–<102  Women: 80–<88  Men: ≥102  Women: ≥88 | Favorable  Favorable  Intermediate  Intermediate  Unfavorable  Unfavorable | 0.5  0.5  0.25  0.25  0  0 |
| 2. Be physically active | Physical activity 10+ min (days/week) |  |  |
|  | Moderate 6-7 or vigorous 3-7  Moderate 1-5 or vigorous 1-2  None | Favorable  Intermediate  Unfavorable | 1  0.5  0 |
| 3. Sedentary time | Sedentary time ^c^(hours/day) |  |  |
|  | < 3  3-4.9  ≥ 5 | Favorable  Intermediate  Unfavorable | 1  0.5  0 |
| 4. Eat a diet rich in whole grains, vegetables, fruit and beans | Fruits and vegetables intake ^d^ |  |  |
|  | ≥5 servings/day  ≥3-<5 servings/day  <3 servings/day | Favorable  Intermediate  Unfavorable | 0.5  0.25  0 |
|  | Whole grains intake ^e^ |  |  |
|  | ≥5.5 servings/day  >2-<5.5 servings/day  ≤2 serving/day | Favorable  Intermediate  Unfavorable | 0.5  0.25  0 |
| 5. Limit consumption of red meat | Red meat |  |  |
|  | ≤2times/week  >2-≤4times/week  >4 times/week | Favorable  Intermediate  Unfavorable | 1  0.5  0 |
| 6. Limit alcohol consumption | Alcohol intake frequency ^f^ |  |  |
|  | Never or seldom  1-4 times per week  Almost daily | Favorable  Intermediate  Unfavorable | 1  0.5  0 |
| 7. Restricted smoking | Smoking |  |  |
|  | Never  Previous  Current | Favorable  Intermediate  Unfavorable | 1  0.5  0 |
| 8. Sleep time | Sleep time (hours/day) |  |  |
|  | 7  6 or 8  ＜5 or ＞8 | Favorable  Intermediate  Unfavorable | 1  0.5  0 |
| Total Score Range |  |  | 0-8 |
| ^a^ The unweighted healthy lifestyle score based on the World Cancer Research Fund/American Institute of Cancer Research (WCRF/AICR) lifestyle score and the American Cancer Society (ACS) Guidelines on Nutrition and Physical Activity for Cancer Prevention.  ^b^ BMI, body mass index.  ^c^ Sedentary time, time spent engaging in sedentary activity (driving, watching TV, computer using).  ^d^ Amount per serving: fresh fruit - 1 piece; dried fruit - 5 pieces; cooked/raw/salad vegetables - 3 heaped tablespoons.  ^e^ Amount per serving: Bran/oat/muesli cereal - 1 bowl/day; Whole-meal/wholegrain bread - 1 slice/day.  ^f^ Seldom, special occasions only, and 1-3 times a month. | | | |

**Table S3 Data fields and information on variables in the UK Biobank cohort involved in this study**

| Category | Data field | Description |
| --- | --- | --- |
| Demographic factors |  |  |
| Age | 21022 | Age at recruitment |
| Sex | 31 | Sex |
| Education level | 6138 | Qualifications |
| Socioeconomic status | 189 | Townsend deprivation index at recruitment |
| Ethnic background | 21000 | Ethnic background |
| Lifestyle factors |  |  |
| BMI | 21001 | Body mass index (BMI) |
| Waist circumference | 48 | Waist circumference |
| Physical activity 10+ min | 884 | Number of days/week of moderate physical activity 10+ minutes |
|  | 904 | Number of days/week of vigorous physical activity 10+ minutes |
| Sedentary time | 1090 | Time spent driving |
|  | 1080 | Time spent using computer |
|  | 1070 | Time spent watching television (TV) |
| Fruit and vegetable intake | 1309 | Fresh fruit intake |
|  | 1319 | Dried fruit intake |
|  | 1289 | Cooked vegetable intake |
|  | 1299 | Salad / raw vegetable intake |
| Whole grains intake | 1458 | Cereal intake |
|  | 1438 | Bread intake |
| Red meat intake | 1369 | Beef intake |
|  | 1379 | Lamb/mutton intake |
|  | 1389 | Pork intake |
| Alcohol intake frequency | 1558 | Alcohol intake frequency |
| Smoking | 20116 | Smoking status |
| Sleep time | 1160 | Sleep duration |
| Other factors |  |  |
| Urate | 30880 | Urate |
| Vascular/heart problems | 6150 | Vascular/heart problems diagnosed by doctor |
| Diabetes | 2443 | Diabetes diagnosed by doctor |

**Table S4 HR for cancers in patients with and without gout (controls) after adjusting for covariates**

| Variable | Total Cancer  HR(95% CI)^a^ | *P* | Global *P*-value | Total Cancer  HR(95% CI)^b^ | *P* | Global *P-*value |
| --- | --- | --- | --- | --- | --- | --- |
| Non-gout | 1.000(ref) |  |  | 1.000(ref) |  |  |
| gout | 1.122(1.060，1.189) | <0.001 | 0.87 | 1.087(1.020，1.158) | 0.009 | 0.87 |

^a^ Model 1 was not adjusted;

^b^ Model 2 was adjusted for age, sex, race, BMI, education level, Thomson index, heart/cardiovascular disease, diabetes mellitus, and urate.

**Table S5 Baseline characteristics of participants in the UK Biobank classified by the presence or absence of cancer**

| Characteristics | | Part A^a^ | | |  | *P* value | Part B^b^ | | |  | *P* value |
| --- | --- | --- | --- | --- | --- | --- | --- | --- | --- | --- | --- |
|  |  | Overall(n=28676) | Non-cancer(n=22519) | Cancer(n=6157) | t/χ^2^ |  | Overall(n=6105) | Non-cancer(n=4752) | Cancer(n=1353) | t/χ^2^ |  |
| Age, years, mean±SD | | 59.5±7.00 | 58.8±7.19 | 62.3±5.40 | -40.86 | <0.001 | 59.4±7.00 | 58.7±7.22 | 62.1±5.41 | -18.78 | <0.001 |
| Sex | |  |  |  | 6.63 | 0.010 |  |  |  | 1.06 | 0.302 |
| Female | | 2024 | 1648 | 376 |  |  | 377 | 302 | 75 |  |  |
| Male | | 26652 | 21051 | 5601 |  |  | 5728 | 4450 | 1278 |  |  |
| Race | |  |  |  | 80.94 | <0.001 |  |  |  | 13.55 | <0.001 |
| White | | 27239 | 21426 | 5813 |  |  | 5862 | 4539 | 1323 |  |  |
| Non-white | | 1437 | 1273 | 164 |  |  | 243 | 213 | 30 |  |  |
| Educational level | |  |  |  | 100.72 | <0.001 |  |  |  | 524.87 | <0.001 |
| College or University | | 8652 | 7038 | 1614 |  |  | 1652 | 1302 | 350 |  |  |
| Upper secondary | | 2771 | 2235 | 536 |  |  | 662 | 542 | 120 |  |  |
| Lower secondary | | 6612 | 5333 | 1279 |  |  | 1584 | 1291 | 293 |  |  |
| Vocational or other | | 10427 | 7925 | 2502 |  |  | 2207 | 1617 | 590 |  |  |
| Socio-economic status | |  |  |  | 2282.40 | <0.001 |  |  |  | 1742.61 | <0.001 |
| Low | | 9535 | 7609 | 1926 |  |  | 2030 | 1599 | 431 |  |  |
| Middle | | 9538 | 7477 | 2061 |  |  | 2036 | 1590 | 446 |  |  |
| High | | 9603 | 7433 | 2170 |  |  | 2039 | 1563 | 476 |  |  |
| Smoking status | |  |  |  | 101.13 | <0.001 |  |  |  | 720.41 | <0.001 |
| Never | | 13390 | 10854 | 2425 |  |  | 2607 | 2128 | 479 |  |  |
| Previous | | 12204 | 9372 | 2832 |  |  | 2950 | 2208 | 742 |  |  |
| Current | | 3041 | 2352 | 689 |  |  | 548 | 416 | 132 |  |  |
| Alcohol intake frequency | |  |  |  | 28.14 | <0.001 |  |  |  | 13.62 | <0.001 |
| Daily or almost daily | | 7981 | 6190 | 1791 |  |  | 2069 | 1574 | 495 |  |  |
| Three or four times a week | | 7480 | 5912 | 1568 |  |  | 1698 | 1321 | 377 |  |  |
| Once or twice a week | | 6930 | 5534 | 1396 |  |  | 1369 | 1077 | 292 |  |  |
| One to three times a month | | 2297 | 1842 | 455 |  |  | 362 | 293 | 69 |  |  |
| Special occasions only | | 2111 | 1681 | 430 |  |  | 311 | 241 | 70 |  |  |
| Never | | 1802 | 1473 | 329 |  |  | 296 | 246 | 50 |  |  |
| BMI, kg/m^2^, mean±SD | | 29.0±4.75 | 29.0±4.81 | 29.0±4.57 | -1.64 | 0.100 | 30.7±4.90 | 30.8±4.95 | 30.3±4.71 | 3.23 | 0.001 |
| Vascular/heart problems | |  |  |  | 102.22 | <0.001 |  |  |  | 1653.29 | <0.001 |
| Heart attack | | 1445 | 1106 | 339 |  |  | 433 | 341 | 92 |  |  |
| Angina | | 1160 | 882 | 278 |  |  | 320 | 242 | 78 |  |  |
| Stroke | | 573 | 411 | 162 |  |  | 168 | 121 | 47 |  |  |
| High blood pressure | | 9239 | 7102 | 2137 |  |  | 2844 | 2164 | 680 |  |  |
| Diabetes | | 2582 | 1972 | 610 | 19.45 | <0.001 | 843 | 634 | 209 | 6.67 | 0.035 |
| Urate, umol/L, mean±SD | | 360.9±83.8 | 361.0±83.9 | 360.0±83.8 | -0.12 | 0.903 | 379.9±102.5 | 383.0±102.0 | 370.0±103.0 | 4.12 | <0.001 |
|  | ^a^ is exploring whether gout can increase cancer risk;  ^b^ is exploring whether a healthy lifestyle in can reduce the increased cancer risk of gout. | | | | | | | | |  |  |

**Table S6 Baseline characteristics of lifestyle factors among gout patients by gender (n=6105)**

| Characteristics | Female(n=377) | Male(n=5,728) | t/χ^2^ value | *P*-value |
| --- | --- | --- | --- | --- |
| Cancer |  |  | 1.06 | 0.302 |
| No | 302 | 4450 |  |  |
| Yes | 75 | 1278 |  |  |
| Age, years, mean±SD | 61.40±5.98 | 59.30±7.04 | 6.65 | <0.001 |
| Race |  |  |  | <0.001 |
| White | 341 | 5521 |  |  |
| Non-white | 36 | 207 |  |  |
| Education level |  |  | 9.51 | 0.023 |
| College or University | 80 | 1572 |  |  |
| Upper secondary | 35 | 627 |  |  |
| Lower secondary | 110 | 1474 |  |  |
| Vocational or other | 152 | 2055 |  |  |
| Socioeconomic status |  |  | 28.85 | <0.001 |
| Low | 167 | 1863 |  |  |
| Middle | 125 | 1911 |  |  |
| High | 85 | 1954 |  |  |
| Urate, mean(sd), umol/L | 338.00±109.00 | 383.00±101.00 | -7.82 | <0.001 |
| Vascular/heart problems |  |  | 43.91 | <0.001 |
| Heart attack | 15 | 418 |  |  |
| Angina | 25 | 295 |  |  |
| Stroke | 18 | 150 |  |  |
| High blood pressure | 223 | 2621 |  |  |
| Diabetes | 77 | 766 | 19.37 | <0.001 |
| BMI(kg/m^2^) |  |  | 34.28 | <0.001 |
| Unfavorable | 234 | 2714 |  |  |
| Intermediate | 110 | 2534 |  |  |
| Favorable | 33 | 480 |  |  |
| Waist circumference (cm) |  |  | 95.35 | <0.001 |
| Unfavorable | 300 | 3091 |  |  |
| Intermediate | 40 | 1582 |  |  |
| Favorable | 37 | 1055 |  |  |
| Physical activity 10+ min (days/week) |  |  | 9.22 | 0.010 |
| Unfavorable | 79 | 891 |  |  |
| Intermediate | 169 | 2561 |  |  |
| Favorable | 129 | 2276 |  |  |
| Sedentary time (hours/day) |  |  | 3.94 | 0.139 |
| Unfavorable | 216 | 3548 |  |  |
| Intermediate | 123 | 1722 |  |  |
| Favorable | 38 | 458 |  |  |
| Fruit and vegetable intake (servings/day) |  |  | 16.05 | <0.001 |
| Unfavorable | 107 | 2175 |  |  |
| Intermediate | 148 | 2101 |  |  |
| Favorable | 122 | 1452 |  |  |
| Whole grains intake (servings/day) |  |  | 42.84 | <0.001 |
| Unfavorable | 180 | 1829 |  |  |
| Intermediate | 193 | 3710 |  |  |
| Favorable | 4 | 189 |  |  |
| Red meat intake (times/week) |  |  | 17.00 | <0.001 |
| Unfavorable | 18 | 481 |  |  |
| Intermediate | 301 | 4689 |  |  |
| Favorable | 58 | 558 |  |  |
| Alcohol intake frequency |  |  | 386.39 | <0.001 |
| Unfavorable | 52 | 2017 |  |  |
| Intermediate | 142 | 2925 |  |  |
| Favorable | 183 | 786 |  |  |
| Smoking |  |  | 22.18 | <0.001 |
| Unfavorable | 36 | 512 |  |  |
| Intermediate | 139 | 2811 |  |  |
| Favorable | 202 | 2405 |  |  |
| Sleep time (hours/day) |  |  | 16.27 | <0.001 |
| Unfavorable | 86 | 864 |  |  |
| Intermediate | 167 | 2738 |  |  |
| Favorable | 124 | 2126 |  |  |

**Table S7 Baseline characteristics of gout patients by unweighted and weighted HLS classification (n=6105)**

| Characteristics | Unweighted healthy lifestyle score | | |  |  | Weighted healthy lifestyle score | | |  |  |
| --- | --- | --- | --- | --- | --- | --- | --- | --- | --- | --- |
|  | low(n=1597) | medium(n=2381) | high(n=2127) | t/χ^2^ | *P* value | low(n=2025) | medium(n=2044) | high(n=2036) | t/χ^2^ | *P* value |
| Cancer |  |  |  | 13.19 | 0.001 |  |  |  | 30.14 | <0.001 |
| No | 1200 | 1849 | 1703 |  |  | 1493 | 1594 | 1665 |  |  |
| Yes | 397 | 532 | 424 |  |  | 532 | 450 | 371 |  |  |
| Age, mean(sd), year | 59.9±6.74 | 59.7±6.91 | 58.8±7.26 | 12.12 | <0.001 | 60.7±6.67 | 60.1±6.57 | 57.6±7.36 | 10.08 | <0.001 |
| Gender |  |  |  | 12.71 | 0.001 |  |  |  | 0.006 | 0.997 |
| Female | 74 | 147 | 156 |  |  | 126 | 125 | 126 |  |  |
| Male | 1523 | 2234 | 1971 |  |  | 1899 | 1919 | 1910 |  |  |
| Race |  |  |  | 164.07 | <0.001 |  |  |  | 13.86 | 0.001 |
| White | 1567 | 2308 | 1987 |  |  | 1959 | 1982 | 1921 |  |  |
| Non-white | 30 | 73 | 140 |  |  | 66 | 62 | 115 |  |  |
| Educational level |  |  |  | 42.78 | <0.001 |  |  |  | 68.95 | <0.001 |
| College or University | 353 | 587 | 712 |  |  | 462 | 564 | 626 |  |  |
| Upper secondary | 182 | 259 | 221 |  |  | 199 | 217 | 246 |  |  |
| Lower secondary | 438 | 635 | 511 |  |  | 530 | 530 | 524 |  |  |
| Vocational or other | 624 | 900 | 683 |  |  | 834 | 733 | 640 |  |  |
| Socio-economic status |  |  |  | 23.37 | <0.001 |  |  |  | 16.24 | <0.001 |
| Low | 589 | 766 | 675 |  |  | 745 | 653 | 632 |  |  |
| Middle | 505 | 784 | 747 |  |  | 664 | 678 | 694 |  |  |
| High | 503 | 831 | 705 |  |  | 616 | 713 | 710 |  |  |
| Vascular/heart problems |  |  |  | 102.98 | <0.001 |  |  |  | 25.89 | <0.001 |
| Heart attack | 137 | 184 | 112 |  |  | 159 | 169 | 105 |  |  |
| Angina | 100 | 110 | 110 |  |  | 106 | 130 | 84 |  |  |
| Stroke | 50 | 71 | 47 |  |  | 71 | 46 | 51 |  |  |
| High blood pressure | 836 | 1112 | 896 |  |  | 946 | 949 | 949 |  |  |
| Diabetes | 281 | 324 | 238 | 38.37 | 0.003 | 272 | 296 | 275 | 5.40 | 0.248 |
| Urate, mean(sd), umol/L | 380.0±106.0 | 381.0±102.0 | 379.0±100.0 | 0.674 | 0.713 | 374.0±104.0 | 379.0±102.0 | 387.0±101.0 | 1.10 | 0.575 |

**Table S8 Results of one-way Cox regression analysis of lifestyle factors and cancer incidence in gout patients stratified by urate level**

| Characteristics | Low(cancer/total:500/2038) | | Medium(cancer/total:449/2032) | | High(cancer/total:404/2035) | |
| --- | --- | --- | --- | --- | --- | --- |
|  | Model 1^a^ | Model 2^b^ | Model 1^a^ | Model 2^b^ | Model 1^a^ | Model 2^b^ |
| BMI (kg/m^2^) |  |  |  |  |  |  |
| Unfavorable | 1.000(ref) | 1.000(ref) | 1.000(ref) | 1.000(ref) | 1.000(ref) | 1.000(ref) |
| Intermediate | 1.130(0.939,1.359) | 1.103(0.911,1.336) | 1.299(1.070,1.576)** | 1.301(1.066,1.589)** | 0.915(0.747,1.122) | 0.866(0.701,1.070) |
| Favorable | 1.170(0.859,1.593) | 1.204(0.875,1.656) | 1.136(0.799,1.615) | 1.071(0.747,1.536) | 0.924(0.626,1.365) | 0.929(0.622,1.387) |
| WC (cm) |  |  |  |  |  |  |
| Unfavorable | 1.000(ref) | 1.000(ref) | 1.000(ref) | 1.000(ref) | 1.000(ref) | 1.000(ref) |
| Intermediate | 1.100(0.891,1.358) | 1.103(0.890,1.367) | 1.181(0.953,1.463) | 1.210(0.972,1.506) | 0.824(0.655,1.036) | 0.842(0.665,1.066) |
| Favorable | 1.209(0.965,1.514) | 1.275(1.009,1.612)* | 1.090,0.847,1.403) | 1.048(0.809,1.359) | 0.733(0.551,0.975)* | 0.783(0.585,1.048) |
| Physical activity 10+ min (days/week) |  |  |  |  |  |  |
| Unfavorable | 1.000(ref) | 1.000(ref) | 1.000(ref) | 1.000(ref) | 1.000(ref) | 1.000(ref) |
| Intermediate | 0.882(0.681,1.142) | 0.867(0.669,1.125) | 1.085(0.818,1.439) | 1.035(0.778,1.377) | 0.842(0.644,1.101) | 0.833(0.635,1.092) |
| Favorable | 1.012(0.782,1.309) | 0.999(0.771,1.296) | 1.163(0.875,1.545) | 1.098(0.824,1.463) | 0.790(0.599,1.042) | 0.752(0.567,0.996)* |
| Sedentary time (hours/day) |  |  |  |  |  |  |
| Unfavorable | 1.000(ref) | 1.000(ref) | 1.000(ref) | 1.000(ref) | 1.000(ref) | 1.000(ref) |
| Intermediate | 1.085(0.894,1.315) | 1.139(0.937,1.384) | 0.777(0.628,0.963)* | 0.813(0.655,1.009) | 0.986(0.795,1.222) | 0.976(0.786,1.213) |
| Favorable | 1.113(0.784,1.578) | 1.143(0.801,1.630) | 0.899(0.646,1.252) | 0.950(0.680,1.328) | 0.736(0.498,1.088) | 0.728(0.490,1.082) |
| Fruit and vegetable intake (servings/day) |  |  |  |  |  |  |
| Unfavorable | 1.000(ref) | 1.000(ref) | 1.000(ref) | 1.000(ref) | 1.000(ref) | 1.000(ref) |
| Intermediate | 0.932(0.757,1.148) | 0.897(0.727,1.107) | 1.096(0.881,1.362) | 0.983(0.790,1.225) | 1.010(0.810,1.258) | 0.860(0.688,1.076) |
| Favorable | 1.074(0.862,1.338) | 1.012(0.810,1.265) | 1.024(0.808,1.296) | 0.909(0.716,1.155) | 0.957(0.737,1.243) | 0.808(0.620,1.064) |
| Whole grains intake (servings/day) |  |  |  |  |  |  |
| Unfavorable | 1.000(ref) | 1.000(ref) | 1.000(ref) | 1.000(ref) | 1.000(ref) | 1.000(ref) |
| Intermediate | 1.020(0.844,1.233) | 0.956(0.789,1.157) | 1.069(0.877,1.305) | 1.020(0.834,1.248) | 1.157(0.934,1.435) | 1.047(0.842,1.302) |
| Favorable | 0.927(0.546,1.575) | 0.853(0.498,1.461) | 0.983(0.578,1.673) | 1.076(0.629,1.838) | 0.891(0.467,1.700) | 0.927(0.484,1.777) |
| Meat intake (times/week) |  |  |  |  |  |  |
| Unfavorable | 1.000（ref） | 1.000（ref） | 1.000（ref） | 1.000（ref） | 1.000(ref) | 1.000（ref） |
| Intermediate | 0.852(0.634,1.147) | 0.933(0.691,1.260) | 0.938(0.666,1.321) | 0.948(0.672,1.337) | 0.939(0.660,1.337) | 1.024(0.717,1.460) |
| Favorable | 0.737(0.487,1.117) | 0.814(0.534,1.240) | 0.779(0.493,1.229) | 0.865(0.545,1.371) | 0.740(0.461,1.187) | 0.881(0.546,1.421) |
| Alcohol intake frequency |  |  |  |  |  |  |
| Unfavorable | 1.000(ref) | 1.000(ref) | 1.000(ref) | 1.000(ref) | 1.000(ref) | 1.000(ref) |
| Intermediate | 0.945(0.780,1.145) | 0.965(0.794,1.172) | 0.869(0.711,1.064) | 0.955(0.778,1.173) | 0.842(0.681,1.041) | 0.936(0.756,1.161) |
| Favorable | 0.778(0.592,1.022) | 0.806(0.606,1.073) | 0.829(0.617,1.116) | 0.990(0.724,1.352) | 0.898(0.662,1.218) | 1.046(0.758,1.443) |
| Smoking |  |  |  |  |  |  |
| Unfavorable | 1.000(ref) | 1.000(ref) | 1.000(ref) | 1.000(ref) | 1.000(ref) | 1.000(ref) |
| Intermediate | 1.118(0.801,1.562) | 0.950(0.676,1.335) | 0.961(0.706,1.307) | 0.756(0.552,1.034) | 1.011(0.730,1.400) | 0.712(0.510,0.995)* |
| Favorable | 0.720(0.507,1.022) | 0.707(0.496,1.008) | 0.711(0.516,0.979)* | 0.682(0.492,0.944)* | 0.714(0.510,0.998)* | 0.651(0.463,0.915)** |
| Sleep time (hours/day) |  |  |  |  |  |  |
| Unfavorable | 1.000(ref) | 1.000(ref) | 1.000(ref) | 1.000(ref) | 1.000(ref) | 1.000(ref) |
| Intermediate | 0.805(0.636,1.020) | 0.868(0.684,1.102) | 1.024(0.777,1.350) | 1.088(0.824,1.437) | 0.844(0.639,1.115) | 0.959(0.721,1.275) |
| Favorable | 0.823(0.641,1.056) | 0.929(0.722,1.197) | 0.896(0.671,1.197) | 1.025(0.764,1.375) | 0.768(0.575,1.026) | 0.952(0.706,1.283) |
| HLS |  |  |  |  |  |  |
| Low | 1.000(ref) | 1.000(ref) | 1.000(ref) | 1.000(ref) | 1.000(ref) | 1.000(ref) |
| Medium | 1.109(0.886,1.388) | 0.901(0.724,1.121) | 1.241(0.977,1.575) | 0.975(0.774,1.227) | 1.687(1.311,2.171) | 0.794(0.628,1.004) |
| High | 1.010(0.820,1.243) | 0.963(0.765,1.211) | 1.183(0.950,1.473) | 0.879(0.689,1.123) | 1.292(1.017,1.642) | 0.643(0.497,0.832)** |
| Weighted HLS |  |  |  |  |  |  |
| Low | 1.000(ref) | 1.000(ref) | 1.000(ref) | 1.000(ref) | 1.000(ref) | 1.000(ref) |
| Medium | 0.773(0.630,0.948)* | 0.821(0.668,1.008) | 0.798(0.642,0.992)* | 0.863(0.692,1.075) | 0.894(0.707,1.129) | 0.896(0.706,1.137) |
| High | 0.636(0.509,0.793)*** | 0.751(0.599,0.941)* | 0.634(0.504,0.798)*** | 0.769(0.607,0.975)* | 0.691(0.543,0.879)** | 0.885(0.690,1.136) |
| ^a^ Model 1 was not adjusted;  ^b^ Model 2 was adjusted for age, sex, race, education level, Thomson index, heart/cardiovascular disease, diabetes mellitus, urate;  **P*<0.05, ***P*<0.01, ****P*<0.001. | | | | | | |

**Table S9 Results of one-way Cox regression analysis of lifestyle factors and cancer incidence in male gout patients**

| Characteristics | cancer/noncancer | Model 1^a^ | | *P* trend | Model 2^b^ | |
| --- | --- | --- | --- | --- | --- | --- |
|  |  | HR(95% CI) | *P-*value |  | HR(95% CI) | *P-*value |
| BMI (kg/m^2^) |  |  |  | 0.093 |  |  |
| Unfavorable | 576/2138 | 1.000(ref) |  |  | 1.000(ref) |  |
| Intermediate | 594/1940 | 1.103(0.983,1.237) | 0.093 |  | 1.067(0.949,1.201) | 0.276 |
| Favorable | 108/372 | 1.084(0.882,1.331) | 0.442 |  | 1.079(0.874,1.330) | 0.477 |
| Waist circumference (cm) |  |  |  | 0.985 |  |  |
| Unfavorable | 685/2406 | 1.000(ref) |  |  | 1.000(ref) |  |
| Intermediate | 354/1228 | 1.001(0.880,1.138) | 0.985 |  | 1.011(0.888,1.151) | 0.866 |
| Favorable | 239/816 | 1.009(0.870,1.169) | 0.908 |  | 1.033,0.888,1.200) | 0.672 |
| Physical activity 10+ min (days/week) |  |  |  | 0.467 |  |  |
| Unfavorable | 203/688 | 1.000(ref) |  |  | 1.000(ref) |  |
| Intermediate | 559/2002 | 0.942(0.802,1.106) | 0.468 |  | 0.928(0.789,1.091) | 0.367 |
| Favorable | 516/1760 | 0.980(0.833,1.153) | 0.811 |  | 0.948(0.805,1.117) | 0.529 |
| Sedentary time (hours/day) |  |  |  | 0.379 |  |  |
| Unfavorable | 811/2737 | 1.000(ref) |  |  | 1.000(ref) |  |
| Intermediate | 377/1345 | 0.946(0.837,1.070) | 0.380 |  | 0.969(0.857,1.096) | 0.626 |
| Favorable | 90/368 | 0.835(0.671,1.039) | 0.105 |  | 0.860(0.690,1.072) | 0.180 |
| Fruit and vegetable intake (servings/day) |  |  |  | 0.697 |  |  |
| Unfavorable | 478/1697 | 1.000(ref) |  |  | 1.000(ref) |  |
| Intermediate | 474/1627 | 1.025(0.903,1.164) | 0.698 |  | 0.921(0.811,1.047) | 0.210 |
| Favorable | 326/1126 | 1.037(0.900,1.194) | 0.615 |  | 0.917(0.796,1.057) | 0.235 |
| Whole grains intake (servings/day) |  |  |  | 0.205 |  |  |
| Unfavorable | 391/1438 | 1.000(ref) |  |  | 1.000(ref) |  |
| Intermediate | 847/2863 | 1.080(0.958,1.218) | 0.206 |  | 1.020(0.904,1.150) | 0.747 |
| Favorable | 40/149 | 0.964(0.696,1.335) | 0.827 |  | 0.983(0.709,1.364) | 0.920 |
| Meat intake (times/week) |  |  |  | 0.255 |  |  |
| Unfavorable | 116/365 | 1.000（ref） |  |  | 1.000(ref) |  |
| Intermediate | 1060/3629 | 0.894(0.738,1.083) | 0.255 |  | 0.947(0.781,1.147) | 0.580 |
| Favorable | 102/456 | 0.721(0.552,0.941) | 0.016 |  | 0.826(0.632,1.080) | 0.163 |
| Alcohol intake frequency |  |  |  | 0.037 |  |  |
| Unfavorable | 485/1532 | 1.000(ref) |  |  | 1.000(ref) |  |
| Intermediate | 639/2286 | 0.882(0.784,0.993) | 0.037 |  | 0.950(0.843,1.070) | 0.404 |
| Favorable | 154/632 | 0.831(0.693,0.996) | 0.046 |  | 0.914(0.759,1.100) | 0.344 |
| Smoking |  |  |  | 0.417 |  |  |
| Unfavorable | 120/392 | 1.000(ref) |  |  | 1.000(ref) |  |
| Intermediate | 712/2099 | 1.083(0.892,1.314) | 0.417 |  | 0.831(0.683,1.011) | 0.065 |
| Favorable | 446/1959 | 0.744(0.608,0.910) | 0.004 |  | 0.706(0.575,0.865) | ＜0.001 |
| Sleep time (hours/day) |  |  |  | 0.070 |  |  |
| Unfavorable | 213/651 | 1.000(ref) |  |  | 1.000(ref) |  |
| Intermediate | 616/2122 | 0.866(0.741,1.012) | 0.070 |  | 0.963(0.823,1.127) | 0.644 |
| Favorable | 449/1677 | 0.802(0.681,0.944) | 0.008 |  | 0.965(0.817,1.139) | 0.678 |
| HLS |  |  |  | 0.085 |  |  |
| Low | 380/1143 | 1.000(ref) |  |  | 1.000(ref) |  |
| Medium | 508/1726 | 0.890(0.779,1.017) | 0.085 |  | 0.907(0.793,1.037) | 0.153 |
| High | 390/1581 | 0.752(0.653,0.867) | ＜0.001 |  | 0.822(0.712,0.949) | 0.007 |
| Weighted HLS |  |  |  | 0.001 |  |  |
| Low | 499/1400 | 1.000(ref) |  |  | 1.000(ref) |  |
| Medium | 425/1494 | 0.814(0.715,0.926) | 0.001 |  | 0.857(0.752,0.976) | 0.020 |
| High | 254/1556 | 0.653(0.569,0.748) | ＜0.001 |  | 0.816(0.710,0.938) | 0.004 |
| ^a^ Model 1 was not adjusted; ^b^ Model 2 was adjusted for age, sex, race, education level, Thomson index, heart/cardiovascular disease, diabetes mellitus, urate. | | | | | | |

**Table S10 Results of one-way Cox regression analysis of lifestyle factors and cancer incidence in female gout patients**

| Characteristics | cancer/noncancer | Model 1^a^ | | *P* trend | Model 2^b^ | |
| --- | --- | --- | --- | --- | --- | --- |
|  |  | HR(95% CI) | *P-*value |  | HR(95% CI) | *P-*value |
| BMI (kg/m^2^) |  |  |  | 0.788 |  |  |
| Unfavorable | 45/189 | 1.000(ref) |  |  | 1.000(ref) |  |
| Intermediate | 23/87 | 1.071(0.648,1.771) | 0.788 |  | 1.132(0.669,1.913) | 0.643 |
| Favorable | 7/26 | 1.184(0.533,2.626) | 0.678 |  | 1.088(0.475,2.487) | 0.842 |
| Waist circumference (cm) |  |  |  | 0.120 |  |  |
| Unfavorable | 57/243 | 1.000(ref) |  |  | 1.000(ref) |  |
| Intermediate | 11/29 | 1.667(0.874,2.181) | 0.121 |  | 2.077(1.059,4.072) | 0.033 |
| Favorable | 7/30 | 0.981(0.447,2.152) | 0.963 |  | 0.972(0.433.2.182) | 0.946 |
| Physical activity 10+ min (days/week) |  |  |  | 0.261 |  |  |
| Unfavorable | 17/62 | 1.000(ref) |  |  | 1.000(ref) |  |
| Intermediate | 28/141 | 0.708(0.387,1.294) | 0.262 |  | 0.655(0.349,1.228) | 0.187 |
| Favorable | 30/99 | 1.025(0.565,1.860) | 0.933 |  | 1.038(0.562,1.917) | 0.904 |
| Sedentary time (hours/day) |  |  |  | 0.767 |  |  |
| Unfavorable | 40/176 | 1.000(ref) |  |  | 1.000(ref) |  |
| Intermediate | 22/101 | 0.924(0.549,1.555) | 0.767 |  | 0.991(0.576,1.703) | 0.973 |
| Favorable | 13/25 | 1.892(1.012,3.540) | 0.045 |  | 2.042(1.025,4.063) | 0.042 |
| Fruit and vegetable intake (servings/day) |  |  |  | 0.590 |  |  |
| Unfavorable | 22/85 | 1.000(ref) |  |  | 1.000(ref) |  |
| Intermediate | 26/122 | 0.855(0.485,1.510) | 0.591 |  | 0.871(0.484,1.566) | 0.645 |
| Favorable | 27/95 | 1.076(0.613,1.891) | 0.797 |  | 1.127(0.620,2.048) | 0.694 |
| Whole grains intake (servings/day) |  |  |  | 0.927 |  |  |
| Unfavorable | 37/143 | 1.000(ref) |  |  | 1.000(ref) |  |
| Intermediate | 38/155 | 0.979(0.622,1.540) | 0.927 |  | 0.787(0.484,1.280) | 0.335 |
| Favorable | 0/4 | - | 0.996 |  | - | 0.995 |
| Meat intake (times/week) |  |  |  | 0.747 |  |  |
| Unfavorable | 3/15 | 1.000（ref） |  |  | 1.000(ref) |  |
| Intermediate | 60/241 | 1.210(0.379,3.858) | 0.747 |  | 1.176(0.358,3.853) | 0.788 |
| Favorable | 12/46 | 1.273(0.359,4.511) | 0.709 |  | 1.335(0.361,4.927) | 0.664 |
| Alcohol intake frequency |  |  |  | 0.860 |  |  |
| Unfavorable | 10/42 | 1.000(ref) |  |  | 1.000(ref) |  |
| Intermediate | 30/112 | 1.066(0.521,2.181) | 0.861 |  | 1.061(0.498,2.256) | 0.878 |
| Favorable | 35/148 | 1.034(0.512,2.088) | 0.926 |  | 1.060(0.493,2.276) | 0.881 |
| Smoking |  |  |  | 0.153 |  |  |
| Unfavorable | 12/24 | 1.000(ref) |  |  | 1.000(ref) |  |
| Intermediate | 30/109 | 0.614(0.314,1.200) | 0.153 |  | 0.498(0.237,1.048) | 0.066 |
| Favorable | 33/169 | 0.448(0.231,0.868) | 0.017 |  | 0.417(0.205,0.856) | 0.017 |
| Sleep time (hours/day) |  |  |  | 0.966 |  |  |
| Unfavorable | 17/69 | 1.000(ref) |  |  | 1.000(ref) |  |
| Intermediate | 34/133 | 0.987(0.551,1.768) | 0.966 |  | 0.848(0.467,1.540) | 0.590 |
| Favorable | 24/100 | 0.936(0.502,1.742) | 0.835 |  | 0.852(0.447,1.622) | 0.626 |
| HLS |  |  |  | 0.172 |  |  |
| Low | 17/57 | 1.000(ref) |  |  | 1.000(ref) |  |
| Medium | 24/123 | 0.648(0.348,1.208) | 0.172 |  | 0.579(0.305,1.101) | 0.096 |
| High | 34/122 | 0.892(0.498,1.597) | 0.701 |  | 0.820(0.441,1.525) | 0.531 |
| Weighted HLS |  |  |  | 0.347 |  |  |
| Low | 33/93 | 1.000(ref) |  |  | 1.000(ref) |  |
| Medium | 25/100 | 0.779(0.463,1.310) | 0.347 |  | 0.727(0.425,1.240) | 0.242 |
| High | 17/109 | 0.495(0.275,0.888) | 0.018 |  | 0.482(0.262,0.887) | 0.019 |
| ^a^ Model 1 was not adjusted; ^b^ Model 2 was adjusted for age, sex, race, education level, Thomson index, heart/cardiovascular disease, diabetes mellitus, urate. | | | | | | |

Given the relatively small subsample size of female gout patients in this study, we recognize that the precision of the statistically significant effects of the healthy lifestyle score (HLS) may be limited. This is reflected in the width of the confidence intervals, which suggests variability in the estimates. While the findings remain statistically significant, the broader confidence intervals indicate that caution is warranted when interpreting these results, as they may be less stable compared to those derived from larger subsamples.

**Table S11 Results of one-way Cox regression analysis of gout patients stratified by age due to lifestyle factors and cancer occurrence**

| Characteristics | <60 years | | | ≥60 years | | |
| --- | --- | --- | --- | --- | --- | --- |
|  | cancer/noncancer | Model 1^a^ | Model 2^b^ | cancer/noncancer | Model 1^a^ | Model 2^b^ |
| BMI (kg/m^2^) |  |  |  |  |  |  |
| Unfavorable | 169/1135 | 1.000(ref) | 1.000(ref) | 452/1192 | 1.000(ref) | 1.000(ref) |
| Intermediate | 150/915 | 1.069(0.858,1.332) | 1.135(0.905,1.424) | 467/1112 | 1.073(0.942,1.221) | 1.058(0.926,1.209) |
| Favorable | 26/174 | 0.997(0.660,1.507) | 1.045(0.686,1.590) | 89/224 | 1.072(0.853,1.345) | 1.107(0.877,1.396) |
| Waist circumference (cm) |  |  |  |  |  |  |
| Unfavorable | 194/1225 | 1.000(ref) | 1.000(ref) | 548/1424 | 1.000(ref) | 1.000(ref) |
| Intermediate | 87/602 | 0.905(0.703,1.166) | 0.954(0.735,1.236) | 278/655 | 1.082(0.937,1.251) | 1.061(0.916,1.229) |
| Favorable | 64/397 | 0.995(0.750,1.321) | 1.106(0.825,1.481) | 182/449 | 1.031(0.871,1.219) | 1.030(0.867,1.223) |
| Physical activity 10+ min (days/week) |  |  |  |  |  |  |
| Unfavorable | 62/372 | 1.000(ref) | 1.000(ref) | 158/378 | 1.000(ref) | 1.000(ref) |
| Intermediate | 157/984 | 0.958(0.714,1.287) | 0.991(0736,1.336) | 430/1159 | 0.878(0.731,1.054) | 0.881(0.733,1.058) |
| Favorable | 126/868 | 0.869(0.641,1.179) | 0.897(0.659,1.222) | 420/991 | 0.984(0.820,1.182) | 0.969(0.806,1.166) |
| Sedentary time (hours/day) |  |  |  |  |  |  |
| Unfavorable | 210/1352 | 1.000(ref) | 1.000(ref) | 641/1561 | 1.000(ref) | 1.000(ref) |
| Intermediate | 109/687 | 1.004(0.796,1.266) | 1.024(0.810,1.294) | 290/759 | 0.941(0.819,1.082) | 0.956(0.831,1.099) |
| Favorable | 26/185 | 0.903(0.600,1.358) | 0.902(0.597,1.362) | 77/208 | 0.899(0.709,1.139) | 0.934(0.735,1.186) |
| Fruit and vegetable intake (servings/day) |  |  |  |  |  |  |
| Unfavorable | 148/946 | 1.000(ref) | 1.000(ref) | 352/836 | 1.000(ref) | 1.000(ref) |
| Intermediate | 116/771 | 0.956(0.750,1.220) | 0.889(0.696,1.136) | 384/978 | 0.934(0.808,1.080) | 0.924(0.799,1.069) |
| Favorable | 81/507 | 1.036(0.790,1.359) | 0.932(0.708,1.228) | 272/714 | 0.915(0.781,1.072) | 0.916(0.781,1.075) |
| Whole grains intake (servings/day) |  |  |  |  |  |  |
| Unfavorable | 122/774 | 1.000(ref) | 1.000(ref) | 306/807 | 1.000(ref) | 1.000(ref) |
| Intermediate | 210/1366 | 0.979(0.783,1.224) | 0.962(0.767,1.207) | 675/1652 | 1.067(0.932,1.221) | 1.021(0.891,1.169) |
| Favorable | 13/84 | 0.958(0.540,1.697) | 0.960(0.538,1.713) | 27/69 | 1.004(0.677,1.488) | 0.929(0.925,1.381) |
| Meat intake (times/week) |  |  |  |  |  |  |
| Unfavorable | 30/155 | 1.000（ref） | 1.000(ref) | 89/225 | 1.000(ref) | 1.000(ref) |
| Intermediate | 275/1812 | 0.780(0.535,1.138) | 0.848(0.580,1.240) | 845/2058 | 0.992(0.789,1.235) | 1.012(0.813,1.260) |
| Favorable | 40/257 | 0.824(0.513,1.324) | 0.881(0.547,1.418) | 74/245 | 0.781(0.574,1.064) | 0.843(0.617,1.151) |
| Alcohol intake frequency |  |  |  |  |  |  |
| Unfavorable | 108/658 | 1.000(ref) | 1.000(ref) | 387/916 | 1.000(ref) | 1.000(ref) |
| Intermediate | 184/1191 | 0.933(0.735,1.184) | 0.979(0.770,1.244) | 485/1207 | 0.947(0.828,1.083) | 0.944(0.825,1.081) |
| Favorable | 53/375 | 0.906(0.652,1.259) | 0.921(0.652,1.301) | 136/405 | 0.879(0.723,1.069) | 0.932(0.759,1.144) |
| Smoking |  |  |  |  |  |  |
| Unfavorable | 43/251 | 1.000(ref) | 1.000(ref) | 89/165 | 1.000(ref) | 1.000(ref) |
| Intermediate | 136/810 | 0.974(0.691,1.373) | 0.855(0.603,1.211) | 606/1398 | 0.903(0.643,1.003) | 0.766(0.611,0.959) |
| Favorable | 166/1163 | 0.830(0.594,1.162) | 0.818(0.581,1.150) | 313/965 | 0.620(0.490,0.784) | 0.616(0.485,0.782) |
| Sleep time (hours/day) |  |  |  |  |  |  |
| Unfavorable | 42/280 | 1.000(ref) | 1.000(ref) | 188/440 | 1.000(ref) | 1.000(ref) |
| Intermediate | 148/1041 | 0.917(0.651,1.292) | 0.959(0.678,1.356) | 502/1214 | 0.937(0.792,1.108) | 0.963(0.814,1.141) |
| Favorable | 155/903 | 1.096(0.779,1.542) | 1.222(0.860,1.734) | 318/874 | 0.837(0.699,1.003) | 0.880(0.733,1.056) |
| HLS |  |  |  |  |  |  |
| Low | 94/531 | 1.000(ref) | 1.000(ref) | 303/669 | 1.000(ref) | 1.000(ref) |
| Medium | 128/850 | 0.850(0.651,1.110) | 0.849(0.649,1.110) | 404/999 | 0.901(0.776,1.045) | 0.901(0.775,1.047) |
| High | 123/843 | 0.825(0.630,1.079) | 0.885(0.673,1.165) | 301/860 | 0.786(0.670,0.922) | 0.812(0.690,0.955) |
| Weighted HLS |  |  |  |  |  |  |
| Low | 104/580 | 1.000(ref) | 1.000(ref) | 428/913 | 1.000(ref) | 1.000(ref) |
| Medium | 104/682 | 0.861(0.656,1.131) | 0.849(0.645,1.118) | 346/912 | 0.834(0.723,0.960) | 0.856(0.742,0.987) |
| High | 137/962 | 0.801(0.620,1.034) | 0.865(0.668,1.121) | 234/703 | 0.734(0.625,0.860) | 0.764(0.651,0.898) |
| ^a^ Model 1 was not adjusted; ^b^ Model 2 was adjusted for age, sex, race, education level, Thomson index, heart/cardiovascular disease, diabetes mellitus, urate. | | | | | | |

**Table S12 Relationship between lifestyle and cancer risk in gout patients after removal of follow-up time of less than 2 years**

| Characteristics | cancer/noncancer | Model 1^a^ | | *P* trend | Model 2^b^ | |
| --- | --- | --- | --- | --- | --- | --- |
|  |  | HR(95% CI) | *P-*value |  | HR(95% CI) | *P-*value |
| BMI (kg/m^2^) |  |  |  | 0.166 |  |  |
| Unfavorable | 571/2317 | 1.000(ref) |  |  | 1.000(ref) |  |
| Intermediate | 565/2014 | 1.100(0.979,1.236) | 0.107 |  | 1.056(0.937,1.190) | 0.370 |
| Favorable | 104/396 | 1.076(0.872,1.326) | 0.494 |  | 1.057(0.854,1.308) | 0.609 |
| Waist circumference (cm) |  |  |  | 0.970 |  |  |
| Unfavorable | 683/2637 | 1.000(ref) |  |  | 1.000(ref) |  |
| Intermediate | 335/1247 | 1.020(0.895,1.163) | 0.763 |  | 1.023(0.895,1.168) | 0.738 |
| Favorable | 222/843 | 0.995(0.855,1.158) | 0.950 |  | 1.005(0.861,1.174) | 0.941 |
| Physical activity 10+ min (days/week) |  |  |  | 0.888 |  |  |
| Unfavorable | 199/742 | 1.000(ref) |  |  | 1.000(ref) |  |
| Intermediate | 543/2131 | 0.947(0.805,1.114) | 0.514 |  | 0.919(0.780,1.082) | 0.311 |
| Favorable | 498/1854 | 0.990(0.840,1.167) | 0.907 |  | 0.944(0.800,1.114) | 0.500 |
| Sedentary time (hours/day) |  |  |  | 0.244 |  |  |
| Unfavorable | 780/2895 | 1.000(ref) |  |  | 1.000(ref) |  |
| Intermediate | 363/1440 | 0.934(0.825,1.059) | 0.293 |  | 0.956(0.843,1.084) | 0.488 |
| Favorable | 97/392 | 0.918(0.744,1.135) | 0.432 |  | 0.952(0.769,1.178) | 0.652 |
| Fruit and vegetable intake (servings/day) |  |  |  | 0.583 |  |  |
| Unfavorable | 459/1774 | 1.000(ref) |  |  | 1.000(ref) |  |
| Intermediate | 456/1736 | 1.007(0.884,1.146) | 0.916 |  | 0.911(0.800,1.038) | 0.164 |
| Favorable | 325/1217 | 1.042(0.904,1.201) | 0.568 |  | 0.930(0.806,1.074) | 0.326 |
| Whole grains intake (servings/day) |  |  |  | 0.657 |  |  |
| Unfavorable | 398/1572 | 1.000(ref) |  |  | 1.000(ref) |  |
| Intermediate | 806/3002 | 1.056(0.936,1.191) | 0.373 |  | 0.987(0.874,1.114) | 0.832 |
| Favorable | 36/153 | 0.910(0.647,1.281) | 0.591 |  | 0.930(0.660,1.312) | 0.681 |
| Meat intake (times/week) |  |  |  | 0.086 |  |  |
| Unfavorable | 105/376 | 1.000（ref） |  |  | 1.000(ref) |  |
| Intermediate | 1027/3851 | 0.932(0.763,1.140) | 0.496 |  | 0.989(0.808,1.209) | 0.914 |
| Favorable | 108/500 | 0.794(0.607,1.039) | 0.093 |  | 0.910(0.694,1.193) | 0.495 |
| Alcohol intake frequency |  |  |  | 0.014 |  |  |
| Unfavorable | 456/1569 | 1.000(ref) |  |  | 1.000(ref) |  |
| Intermediate | 611/2388 | 0.875(0.775,0.988) | 0.031 |  | 0.940(0.831,1.062) | 0.324 |
| Favorable | 173/770 | 0.829(0.696,0.988) | 0.036 |  | 0.923(0.768,1.109) | 0.393 |
| Smoking |  |  |  | ＜0.001 |  |  |
| Unfavorable | 121/414 | 1.000(ref) |  |  | 1.000(ref) |  |
| Intermediate | 681/2194 | 1.044(0.860,1.266) | 0.662 |  | 0.804(0.661,0.979) | 0.030 |
| Favorable | 438/2119 | 0.710(0.581,0.869) | ＜0.001 |  | 0.667(0.544,0.818) | <0.001 |
| Sleep time (hours/day) |  |  |  | 0.013 |  |  |
| Unfavorable | 207/713 | 1.000(ref) |  |  | 1.000(ref) |  |
| Intermediate | 604/2244 | 0.902(0.770,1.056) | 0.200 |  | 0.981(0.837,1.150) | 0.819 |
| Favorable | 429/1770 | 0.815(0.691,0.963) | 0.016 |  | 0.954(0.806,1.129) | 0.586 |
| HLS |  |  |  | ＜0.001 |  |  |
| Low | 363/1191 | 1.000(ref) |  |  | 1.000(ref) |  |
| Medium | 491/1837 | 0.880(0.768,1.008) | 0.065 |  | 0.891(0.778,1.022) | 0.100 |
| High | 386/1699 | 0.754(0.654,0.871) | ＜0.001 |  | 0.812(0.701,0.939) | 0.005 |
| Weighted HLS |  |  |  | ＜0.001 |  |  |
| Low | 486/1483 | 1.000(ref) |  |  | 1.000(ref) |  |
| Medium | 409/1587 | 0.806(0.706,0.919) | 0.001 |  | 0.848(0.743,0.968) | 0.014 |
| High | 345/1657 | 0.651(0.567,0.747) | ＜0.001 |  | 0.799(0.694,0.920) | 0.001 |
| ^a^ Model 1 was not adjusted; ^b^ Model 2 was adjusted for age, sex, race, education level, Thomson index, heart/cardiovascular disease, diabetes mellitus, urate. | | | | | | |

**Table S13 Results of competing risk regression analyses of lifestyle and cancer risk in gout patients**

| Characteristic | Cancer events | Death events | Model 1^a^ | | Model 2^b^ | |
| --- | --- | --- | --- | --- | --- | --- |
|  |  |  | HR(95% CI) | *P-*value | HR(95% CI) | *P-*value |
| BMI (kg/m^2^) |  |  |  |  |  |  |
| Unfavorable | 621 | 552 | 1.000(ref) |  | 1.000(ref) |  |
| Intermediate | 617 | 371 | 0.966(0.918,1.020) | 0.180 | 1.077(0.965,1.202) | 0.180 |
| Favorable | 115 | 96 | 1.030(0.936,1.130) | 0.570 | 1.018(0.838,1.237) | 0.860 |
| Waist circumference (cm) |  |  |  |  |  |  |
| Unfavorable | 742 | 636 | 1.000(ref) |  | 1.000(ref) |  |
| Intermediate | 365 | 227 | 1.010(0.953,1.070) | 0.780 | 1.023(0.906,1.155) | 0.720 |
| Favorable | 246 | 156 | 0.971(0.911,1.030) | 0.360 | 1.038(0.901,1.196) | 0.600 |
| Physical activity 10+ min (days/week) |  |  |  |  |  |  |
| Unfavorable | 220 | 217 | 1.000(ref) |  | 1.000(ref) |  |
| Intermediate | 587 | 436 | 0.969(0.921,1.020) | 0.220 | 0.946(0.849,1.054) | 0.310 |
| Favorable | 546 | 366 | 1.050(0.999,1.110) | 0.053 | 1.027(0.921,1.146) | 0.630 |
| Sedentary time (hours/day) |  |  |  |  |  |  |
| Unfavorable | 851 | 679 | 1.000(ref) |  | 1.000(ref) |  |
| Intermediate | 399 | 274 | 0.948(0.898,1.000) | 0.055 | 0.985(0.875,1.109) | 0.800 |
| Favorable | 103 | 66 | 0.954(0.872,1.040) | 0.310 | 0.943(0.769,1.155) | 0.570 |
| Fruit and vegetable intake (servings/day) |  |  |  |  |  |  |
| Unfavorable | 500 | 391 | 1.000(ref) |  | 1.000(ref) |  |
| Intermediate | 500 | 356 | 0.986(0.936,1.040) | 0.580 | 0.953(0.853,1.064) | 0.390 |
| Favorable | 353 | 272 | 1.090(1.020,1.150) | 0.005 | 0.970(0.858,1.096) | 0.620 |
| Whole grains intake (servings/day) |  |  |  |  |  |  |
| Unfavorable | 428 | 325 | 1.000(ref) |  | 1.000(ref) |  |
| Intermediate | 885 | 667 | 1.080(0.966,1.210) | 0.180 | 1.013(0.905,1.134) | 0.820 |
| Favorable | 40 | 27 | 0.904(0.662,1.230) | 0.530 | 0.963(0.706,1.314) | 0.810 |
| Meat intake (times/week) |  |  |  |  |  |  |
| Unfavorable | 119 | 103 | 1.000（ref） |  | 1.000(ref) |  |
| Intermediate | 1120 | 816 | 0.938(0.879,1.000) | 0.057 | 1.048(0.909,1.209) | 0.520 |
| Favorable | 114 | 100 | 1.080(0.991,1.170) | 0.080 | 0.884(0.728,1.073) | 0.210 |
| Alcohol intake frequency |  |  |  |  |  |  |
| Unfavorable | 495 | 349 | 1.000(ref) |  | 1.000(ref) |  |
| Intermediate | 669 | 448 | 0.912(0.868,0.959) | <0.001 | 0.999(0.897,1.113) | 0.990 |
| Favorable | 189 | 222 | 0.912(0.868,0.959) | <0.001 | 0.900(0.764,1.060) | 0.210 |
| Smoking |  |  |  |  |  |  |
| Unfavorable | 132 | 132 | 1.000(ref) |  | 1.000(ref) |  |
| Intermediate | 742 | 577 | 1.140(1.080,1.200) | <0.001 | 1.105(0.990,1.233) | 0.074 |
| Favorable | 479 | 310 | 0.877(0.834,0.922) | <0.001 | 0.826(0.737,0.925) | <0.001 |
| Sleep time (hours/day) |  |  |  |  |  |  |
| Unfavorable | 230 | 230 | 1.000(ref) |  | 1.000(ref) |  |
| Intermediate | 650 | 485 | 0.986(0.938,1.040) | 0.570 | 0.987(0.886,1.098) | 0.800 |
| Favorable | 473 | 304 | 0.959(0.911,1.010) | 0.110 | 1.007(0.899,1.128) | 0.910 |
| HLS |  |  |  |  |  |  |
| Low | 397 | 362 | 1.000(ref) |  | 1.000(ref) |  |
| Medium | 532 | 393 | 0.997(0.947,1.050) | 0.900 | 0.990(0.887,1.105) | 0.860 |
| High | 424 | 264 | 0.961(0.913,1.010) | 0.130 | 0.901(0.801,1.014) | 0.085 |
| Weighted HLS |  |  |  |  |  |  |
| Low | 532 | 447 | 1.000(ref) |  | 1.000(ref) |  |
| Medium | 450 | 325 | 1.030(0.976,1.090) | 0.290 | 0.951(0.849,1.066) | 0.390 |
| High | 371 | 247 | 0.894(0.849,0.941) | <0.001 | 0.866(0.766,0.978) | 0.021 |
| ^a^ Model 1 was not adjusted; ^b^ Model 2 was adjusted for age, sex, race, education level, Thomson index, heart/cardiovascular disease, diabetes mellitus, urate. | | | | | | |

**Table S14 Impact of HLS and weighted HLS on survival time at short-, medium- and long-term follow-up times**

| Survival time | Characteristic | | |  | Model 1 ^a^ | | Model 2 ^b^ | |
| --- | --- | --- | --- | --- | --- | --- | --- | --- |
|  |  |  |  | cancer/noncancer | HR(95% CI) | *P*-value | HR(95% CI) | *P*-value |
| ≤5 years | HLS | | Low | 92/46 | 1.000(ref) |  | 1.000(ref) |  |
|  |  |  | Medium | 146/43 | 1.074(0.826,1.394) | 0.594 | 1.073(0.821,1.404) | 0.604 |
|  |  |  | High | 129/26 | 1.224(0.936,1.602) | 0.139 | 1.247(0.939,1.657) | 0.126 |
|  | Weighted HLS | | Low | 136/51 | 1.000(ref) |  | 1.000(ref) |  |
|  |  |  | Medium | 132/37 | 1.271(0.997,1.619) | 0.052 | 1.249(0.973,1.604) | 0.080 |
|  |  |  | High | 99/27 | 1.077(0.829,1.397) | 0.578 | 1.056(0.806,1.383) | 0.690 |
| ≤10 years | HLS | | Low | 164/56 | 1.000(ref) |  | 1.000(ref) |  |
|  |  |  | Medium | 216/59 | 1.223(1.041,1.438) | 0.014 | 1.199(1.017,1.412) | 0.029 |
|  |  |  | High | 152/41 | 1.374(1.158,1.630) | <0.001 | 1.349(1.129,1.612) | <0.001 |
|  | Weighted HLS | | Low | 218/61 | 1.000(ref) |  | 1.000(ref) |  |
|  |  |  | Medium | 167/61 | 1.040(0.891,1.213) | 0.620 | 1.037(0.887,1.212) | 0.643 |
|  |  |  | High | 147/34 | 1.143(0.970,1.345) | 0.109 | 1.137(0.962,1.344) | 0.131 |
| ≤15 years | HLS | | Low | 140/1042 | 1.000(ref) |  | 1.000(ref) |  |
|  |  |  | Medium | 170/1655 | 0.879(0.772,1.001) | 0.052 | 0.903(0.792,1.029) | 0.127 |
|  |  |  | High | 143/1560 | 0.761(0.663,0.873) | <0.001 | 0.830(0.722,0.955) | 0.009 |
|  | Weighted HLS | | Low | 178/1304 | 1.000(ref) |  | 1.000(ref) |  |
|  |  |  | Medium | 150/1429 | 0.802(0.708,0.910) | <0.001 | 0.850(0.749,0.964) | 0.011 |
|  |  |  | High | 125/1524 | 0.641(0.561,0.732) | <0.001 | 0.785(0.686,0.899) | <0.001 |
|  | | a Model 1 was not adjusted;  b Model 2 was adjusted for age, sex, race, education level, Thomson index, heart/cardiovascular disease, diabetes mellitus, urate. | | | | | | |

**Table S15 Number and percentage of missing values in interpolated data variables**

| Variable | Missing number (percentage) |
| --- | --- |
| Ethnic | 42(0.14%) |
| Qualification | 225(0.78%) |
| Townsend deprivation index | 30(0.10%) |
| Household income | 374(1.30%) |
| BMI | 214(0.74%) |
| Urate | 1811(6.31%) |
| Smoke | 42(0.14%) |
| Alcohol | 42(0.14%) |

**Table S16. PCA-Derived HLS Component Weights**

| **Variable** | **PCA Weight** |
| --- | --- |
| BMI Score | **29.4%** |
| Waist Score | **29.5%** |
| Screen Time Score | **12.9%** |
| Activity Score | **9.8%** |
| Alcohol Score | **9.0%** |
| Sleep Score | **4.3%** |
| Meat Score | **3.9%** |
| Fruit/Vegetable/Grain Score | **1.1%** |
| Smoking Score | **0.1%** |

**
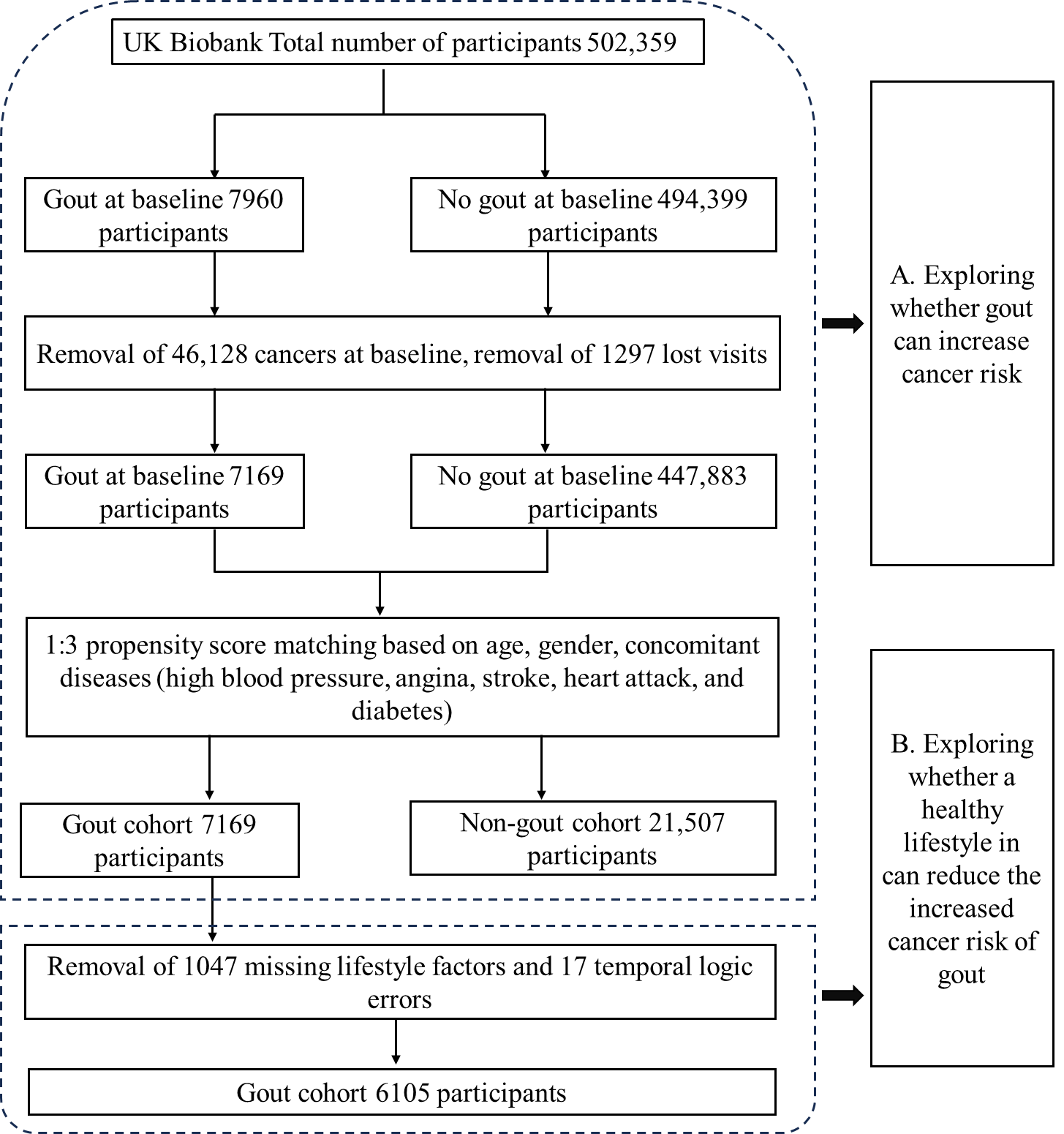
**

**Figure S1 Flowchart of Participant Selection**


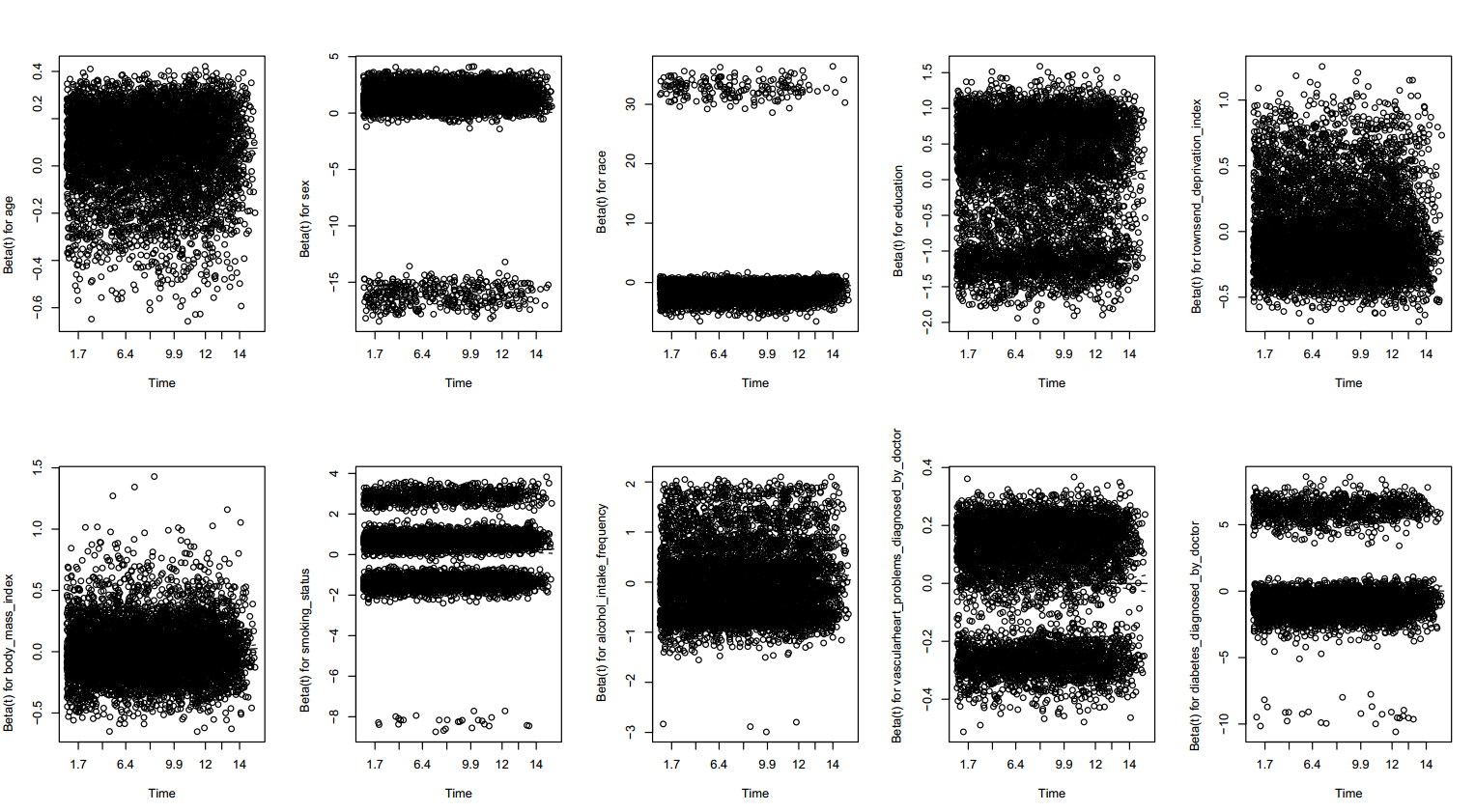


**Figure S2 Schoenfeld residual plot**


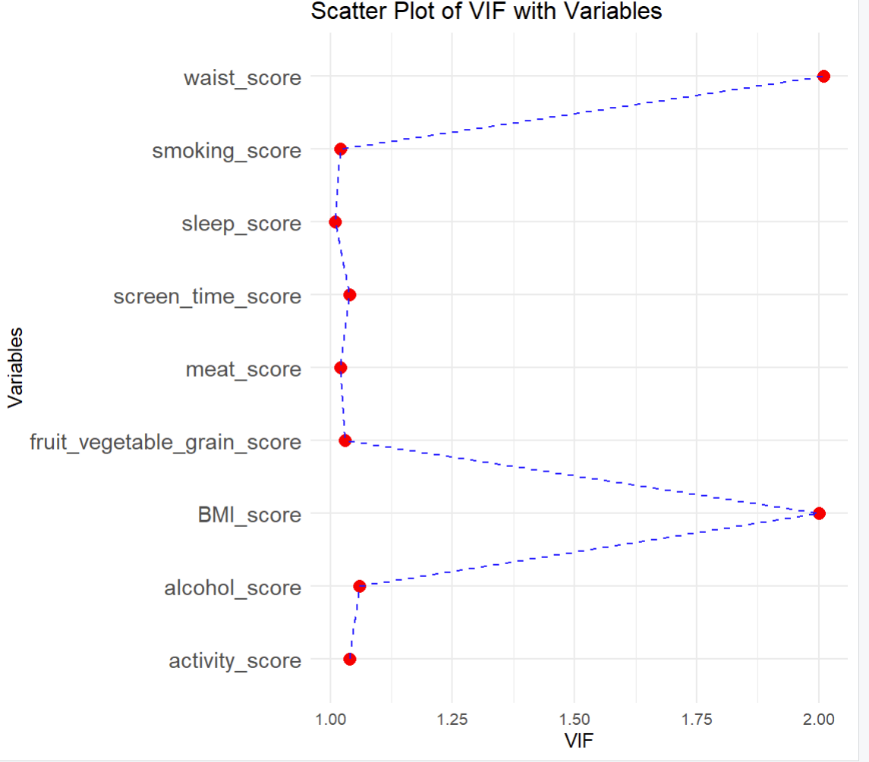


**Figure S3 Scatter Plot of VlF with Variables**
